# A sphingolipid rheostat controls apoptosis versus apical cell extrusion as alternative tumour-suppressive mechanisms

**DOI:** 10.1038/s41419-024-07134-2

**Published:** 2024-10-14

**Authors:** Joy Armistead, Sebastian Höpfl, Pierre Goldhausen, Andrea Müller-Hartmann, Evelin Fahle, Julia Hatzold, Rainer Franzen, Susanne Brodesser, Nicole E. Radde, Matthias Hammerschmidt

**Affiliations:** 1 Institute of Zoology / Developmental Biology, University of Cologne, Cologne, Germany; 2 Center for Molecular Medicine Cologne (CMMC), University of Cologne, Cologne, Germany; 3 Institute for Stochastics and Applications, University of Stuttgart, Stuttgart, Germany; 4 Max-Planck Institute for Plant Breeding Research, Cologne, Germany; 5 Lipidomics/Metabolomics Facility, Cluster of Excellence Cellular Stress Responses in Aging-associated Diseases (CECAD), University of Cologne, Cologne, Germany

**Keywords:** Apoptosis, Disease model, Tumour-suppressor proteins

## Abstract

Evasion of cell death is a hallmark of cancer, and consequently the induction of cell death is a common strategy in cancer treatment. However, the molecular mechanisms regulating different types of cell death are poorly understood. We have formerly shown that in the epidermis of hypomorphic zebrafish *hai1a* mutant embryos, pre-neoplastic transformations of keratinocytes caused by unrestrained activity of the type II transmembrane serine protease Matriptase-1 heal spontaneously. This healing is driven by Matriptase-dependent increased sphingosine kinase (SphK) activity and sphingosine-1-phosphate (S1P)-mediated keratinocyte loss via apical cell extrusion. In contrast, amorphic *hai1a*^*fr26*^ mutants with even higher Matriptase-1 and SphK activity die within a few days. Here we show that this lethality is not due to epidermal carcinogenesis, but to aberrant tp53-independent apoptosis of keratinocytes caused by increased levels of pro-apoptotic C_16_ ceramides, sphingolipid counterparts to S1P within the sphingolipid rheostat, which severely compromises the epidermal barrier. Mathematical modelling of sphingolipid rheostat homeostasis, combined with in vivo manipulations of components of the rheostat or the ceramide de novo synthesis pathway, indicate that this unexpected overproduction of ceramides is caused by a negative feedback loop sensing ceramide levels and controlling ceramide replenishment via de novo synthesis. Therefore, despite their initial decrease due to increased conversion to S1P, ceramides eventually reach cell death-inducing levels, making transformed pre-neoplastic keratinocytes die even before they are extruded, thereby abrogating the normally barrier-preserving mode of apical live cell extrusion. Our results offer an in vivo perspective of the dynamics of sphingolipid homeostasis and its relevance for epithelial cell survival versus cell death, linking apical cell extrusion and apoptosis. Implications for human carcinomas and their treatments are discussed.

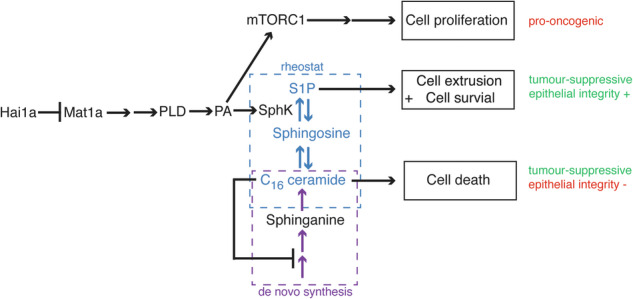

## Introduction

Resisting cell death is one of the hallmarks of cancer [1], thus new chemotherapies are being developed which aim to specifically activate cell death-promoting pathways [2–7]. Therefore, there is a need to understand how cell death is controlled, and how a cell decides to either survive and proliferate or to commit to cell death.

Cell death by apoptosis is induced by the activation of death receptors on the cell surface (extrinsic pathway), or by the activation of a mitochondrial pathway due to cell stress (intrinsic pathway). When the mitochondrial outer membrane is permeabilized, intermembrane proteins including cytochrome c are released, which causes the assembly of the caspase-activating apoptosome. The caspase cysteine protease cascade is initiated, including activation of the executioner caspase 3, resulting in the cleavage of cellular proteins and death of the cell. In multicellular organisms, apoptotic cell corpses are generally cleared by immune cells in a process known as efferocytosis [8, 9]; however, when efferocytosis does not occur, apoptotic cells can undergo secondary necrosis, losing cytoplasmic membrane integrity and releasing damage-associated molecular patterns (DAMPs) [10, 11].

Apical cell extrusion (ACE) is a type of cell loss which occurs in epithelia whereby a cell is squeezed out of the epithelium by its neighbours. This can happen to cells destined for apoptosis [12], or to live cells after neoplastic transformations [13, 14] or upon developmental cell crowding [15] to suppress tumour formation and to maintain epithelial homeostasis. Remarkably, upon cell loss by ACE, epithelial cells seal behind the departing extruded cell, allowing the epithelial barrier to remain intact [11, 12]. Furthermore, in case of extrusion of cells otherwise destined for apoptosis, induction of acute inflammatory responses by such dying cells is prevented [16].

ACE in epithelia can be induced by the action of a bioactive signalling lipid, sphingosine-1-phosphate (S1P), which is released by the departing cell and perceived by cell-surface G-protein coupled S1P receptors on neighbouring cells [17]. In addition, S1P has a (cell-autonomous) pro-cell survival effect, assuring that cells remain alive while being extruded [16]. In other contexts, this pro-survival effect of S1P has been proposed to be achieved by opposing the pro-apoptotic effects of the related sphingolipid ceramide, involving Bax-dependent mitochondrial outer membrane permeabilization (MOMP), cytochrome c release and caspase cascade activation [18–20]. The interconversion of ceramide, sphingosine, and S1P by a complex series of enzymatic reactions is known as the sphingolipid rheostat [21–25], and perturbations of the rheostat can dictate whether a cell lives or dies.

We and others have formerly shown that in zebrafish embryos, loss-of-function mutations in the *hai1a* gene (also known as *spint1a)*, and thereby gain-of-function of its normally inhibited direct substrate, the epithelial cell-surface serine protease Matriptase-1 (also known as ST14), lead to aberrant activation of an epidermal growth factor receptor (EGFR), phospholipase D (PLD), and mechanistic target of rapamycin (mTOR)-dependent signalling cascade that culminates in pre-neoplastic epidermal hyperplasia [26–29]. In parallel, de-regulated Matriptase activity leads to translocation of cytoplasmic sphingosine kinase (SphK) to the plasma membrane, triggering increased S1P production and live cell apical extrusion of overcrowded outer peridermal cells as a counteractive tumour-suppressive branch of the pathway. In the hypomorphic *hai1a*^*hi2217*^ allele that has retained some Hai1a activity, this leads to spontaneous healing of the epidermis and survival of the fish [29]. In contrast, the amorphic *hai1a*^*fr26*^ allele, which displays a complete loss of Hai1a activity, is normally lethal. However, it can also be healed upon sequential treatment with a pharmacological PLD inhibitor and S1P [29].

Here, comparing the viable hypomorphic and the lethal amorphic *hai1a* alleles in more detail, we unravel the mechanisms underlying the lethality of the amorphic *hai1a*^*fr26*^ allele and the rescuing effect of S1P. We identify temporally distinct perturbations in the sphingolipid rheostat in the amorphic allele downstream of increased Matriptase activity which ultimately lead to an over-production of pro-apoptotic ceramides, causing apoptotic cell death in the epidermis as well as loss of epidermal barrier function and death of the organism. Using a systems biology approach to model the activity of the sphingolipid rheostat, we find that the ceramide upregulation cannot be explained by a simple Matriptase-induced increased flux of the sphingolipid rheostat towards S1P. We uncover a negative feedback mechanism which senses deviations from normal sphingolipid levels and counterbalances them by promoting ceramide de novo synthesis. Accordingly, genetic inhibition of ceramide synthase (*cers*) genes or ceramide-promoting elements within the ceramide—S1P rheostat in vivo blocks peridermal apoptosis, thereby restoring the barrier function of the epidermis and extending the life span of the fish. Our results help to explain why, despite the generally accepted tumour-suppressive effects of ceramides [30], elevated CerS levels worsen, rather than improve, the prognosis of different carcinoma types in human [31, 32].

## Results

### Epidermal cells of hai1a^fr26^ mutants undergo cell death around 4 dpf

**Fig. 1:**
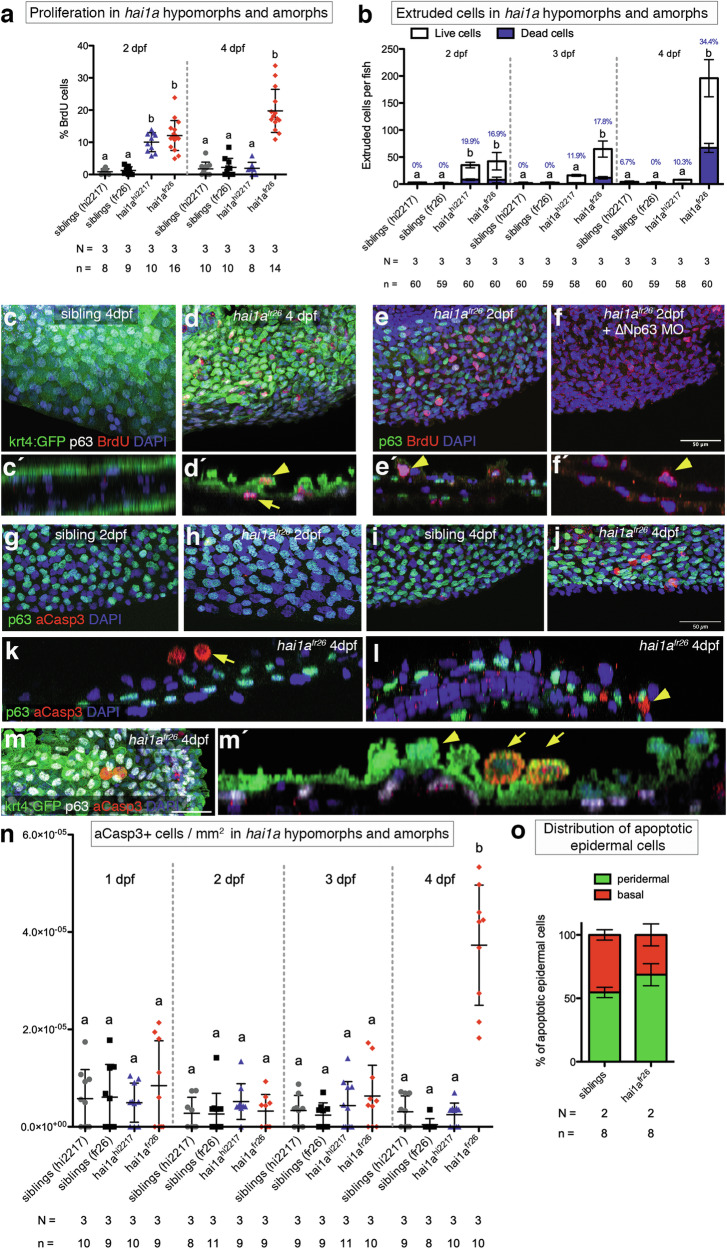
Comparison of proliferation, apical cell extrusion, and apoptosis phenotypes in the *hai1a*^*hi2217*^ hypomorphic and the *hai1a*^*fr26*^ amorphic alleles. **a** Comparison of proliferation rates in the *hai1a*^*hi2217*^ hypomorphic and the *hai1a*^*fr26*^ amorphic alleles at 2 and 4 dpf, relative to the total number of cells. **b** Quantification of extruded epidermal cells in the *hai1a*^*hi2217*^ hypomorphic and the *hai1a*^*fr26*^ amorphic alleles at 2, 3 and 4 dpf, collected from the embryo growth medium. White bars show the numbers of live cells, blue bars show the number of dead cells, with the proportion of dead cells in each condition indicated by blue text. **c**, **d** Representative images of proliferation assay in sibling (**c**) or the *hai1a*^*fr26*^ mutant (**d**) epidermis at 4 dpf in the caudal fin fold with orthogonal views (**c**’, **d**’), showing BrdU-positive cells (red) within the peridermal cell layer (green, yellow arrowhead), or the basal cell layer (white, yellow arrow). **e**, **f** Proliferation assay in *hai1a*^*fr26*^ mutant caudal fin fold epidermis without (**e**) and with (**f**) knockdown of ∆Np63 by antisense morpholino oligonucleotide (MO) to eliminate basal keratinocytes, with orthogonal views in the lower panels (**e**’, **f**’) showing BrdU-positive peridermal cells (yellow arrowheads). BrdU, red; p63, green; nuclei, blue. Scale bar = 50 μm. **g**–**j** Representative images of cleaved caspase 3 (aCasp3)-positive cells on the ventral median fin fold at 2 dpf (**g**, **h**) and 4 dpf (**i**, **j**), sibling (**g**, **i**) or *hai1a*^*fr26*^ (**h**, **j**) mutant epidermis. aCasp3, red; p63, green; nuclei, blue. Scale bar = 50 μm. **k**, **l** Orthogonal views of the epidermis of the 4 dpf mutant fish shown in (**j**) displaying apoptotic cells protruding above the epidermis (**k**), and within the basal layer (**l**). aCasp3, red; p63, green; nuclei, blue. **m** Representative image of aCasp3-positive cells in the *hai1a*^*fr26*^ mutant at 4 dpf in the caudal fin fold with orthogonal view (m’), showing extruding live peridermal cells (green, yellow arrowhead) or extruding aCasp3-positive (red) peridermal cells (green, yellow arrows). Basal cell layer, white. Scale bar = 50 μm. **n** Quantification of the number of aCasp3-positive cells in the tail fin of sibling control, *hai1a*^*hi2217*^, and *hai1a*^*fr26*^ mutant embryos at 1, 2, 3, and 4 dpf, normalised to fin area. **o** Distributions of apoptotic cells within the epidermis in siblings and *hai1a*^*fr26*^ mutants at 4 dpf. For all graphs, means within each time point were compared using a one-way ANOVA with post-hoc Tukey’s multiple comparison test. *N* = number of biological replicates, *n* = number of fish per condition.

Despite activating the same signalling pathways downstream of Matriptase-1, the zebrafish hypomorphic *hai1a*^*hi2217*^ mutant embryo phenotype ultimately resolves completely after 4 days post fertilization (dpf) in a manner dependent on ACE, while the amorphic allele *hai1a*^*fr26*^ is unable to heal and embryos die between 4 and 8 dpf [29]. To look into possible reasons behind the lethality of the amorphic allele, we performed comparative epidermal cell proliferation, ACE and cell death analyses. While at 2 dpf, both proliferation and extrusion rates are similarly increased in the hypomorphic and the amorphic alleles compared to controls, by 4 dpf, both rates have normalized again in the hypomorphic allele [29]. In contrast, in the amorphic allele, both rates have further increased (Fig. 1a, b). Yet, the increase of these counteractive cell gain and cell loss rates in the amorphic allele are rather proportional, and should thus not preclude an eventual healing. Also, as formerly shown for the hypomorphic allele [28], hyperproliferation assessed by bromodeoxyuridine (BrdU) incorporation occurs both in the outer peridermal layer and the inner basal layer of the epidermis of amorphic *hai1a*^*fr26*^ mutants (Fig. 1c, d), and peridermal hyperproliferation even takes place when the proliferation of basal cells is blocked via a ∆Np63 antisense morpholino oligonucleotide (MO) [33, 34] (Fig. 1e, f). This points to a layer-autonomous hyperplastic effect in the periderm, and is in line with the periderm being the primary site of ACE as a tumour-suppressive effect [29]. Yet, these features are also shared between the hypomorphic and amorphic allele and are unlikely to account for the specific lethality of the amorphic allele.

However, crucial differences between the two alleles were found for epidermal cell death rates determined via cleaved caspase 3 (aCasp3) immunofluorescence and terminal deoxynucleotidyl transferase dUTP nick-end labelling (TUNEL). *hai1a*^*hi2217*^ hypomorphic embryos display low cell death rates indistinguishable from those of their siblings at all investigated stages between 2 dpf [29] and 4 dpf (Fig. 1n and Supplementary Fig. S1a–h). Amorphic *hai1a*^*fr26*^ mutant embryos also display unaltered numbers of aCasp3- and TUNEL-positive cells at 1 dpf, 2 dpf, and 3 dpf. However, at 4 dpf, shortly before the mutant embryos themselves begin dying, the number of apoptotic epidermal cells is strongly increased compared to wild-type siblings (Fig. 1g–j, n and S1i-x). Of note, while in control embryos the few apoptotic epidermal cells are relatively equally distributed between the peridermal and the basal layer, the majority (68.4%) of apoptotic cells in *hai1a*^*fr26*^ mutants are in the peridermal layer (Fig. 1o), either still integrated in the tissue or slightly above it and most likely in the process of apical extrusion (Fig. 1k, m). However, the majority of extruding peridermal cells are non-apoptotic (Fig. 1m), in agreement with ACE rates at 4 dpf (Fig. 1b). Yet, apoptosis rates are also highly up-regulated in the basal layer of *hai1a*^*fr26*^ mutants compared to sibling embryos (Fig. 1l).

### Cell death in hai1a^fr26^ mutants is caspase-dependent and p53-independent

**Fig. 2:**
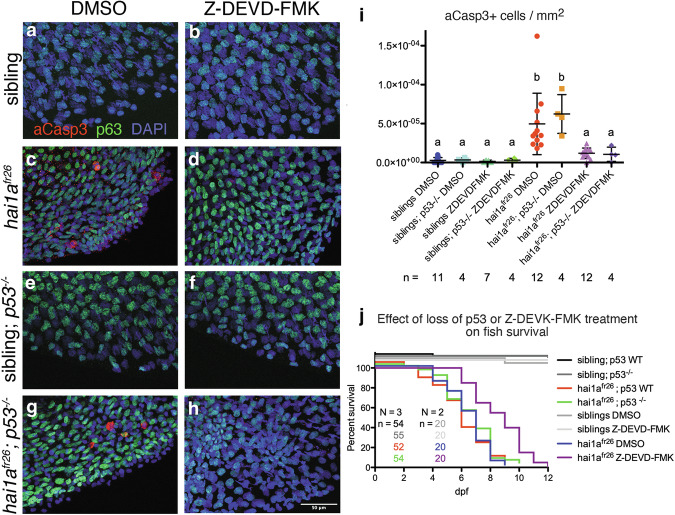
Cell death in *hai1a*^*fr26*^ mutants is caspase-dependent and tp53-independent. **a**–**h** Apoptotic cells in the caudal fin fold of 4 dpf embryos, immunostained for aCasp3 (red), p63 (green) and DAPI (blue), with *tp53* mutation or upon treatment with ZDEVD-FMK to inhibit apoptosis. Scale bar = 50 μm. **i** Quantification of the numbers of aCasp3-positive cells in the tail fins of embryos, normalised to fin area. Means were compared using one-way ANOVA with post-hoc Tukey’s multiple comparison test. **j** Survival curves showing the effects of *tp53* mutation or ZDEVD-FMK treatment on embryo viability.

To confirm that cell death in the amorphic allele is caspase-dependent, embryos were treated with the caspase 3 inhibitor ZDEVD-FMK, which significantly reduces the number of apoptotic cells (Fig. 2a–i). Inhibition of apoptosis also extends the lifespan of *hai1a*^*fr26*^ embryos by almost 4 days on average (Fig. 2a–d, i, j), indicating that the increased epidermal apoptosis found only in the amorphic allele likely contributes to embryo death. On the other hand, knock out of the master cell death regulator tp53 in *hai1a*^*fr26*^;*tp53*^*zdf1*^ double mutants neither changes the number of apoptotic cells nor alters embryo lifespan (Fig. 2a, c, e, g, i, j). Thus, epidermal cell death in *hai1a*^*fr26*^ mutants is a p53-independent, caspase-3 dependent apoptosis, and coincides with embryonic lethality.

### Sphingosine-1-phosphate alleviates cell death in hai1a mutant epidermis

**Fig. 3:**
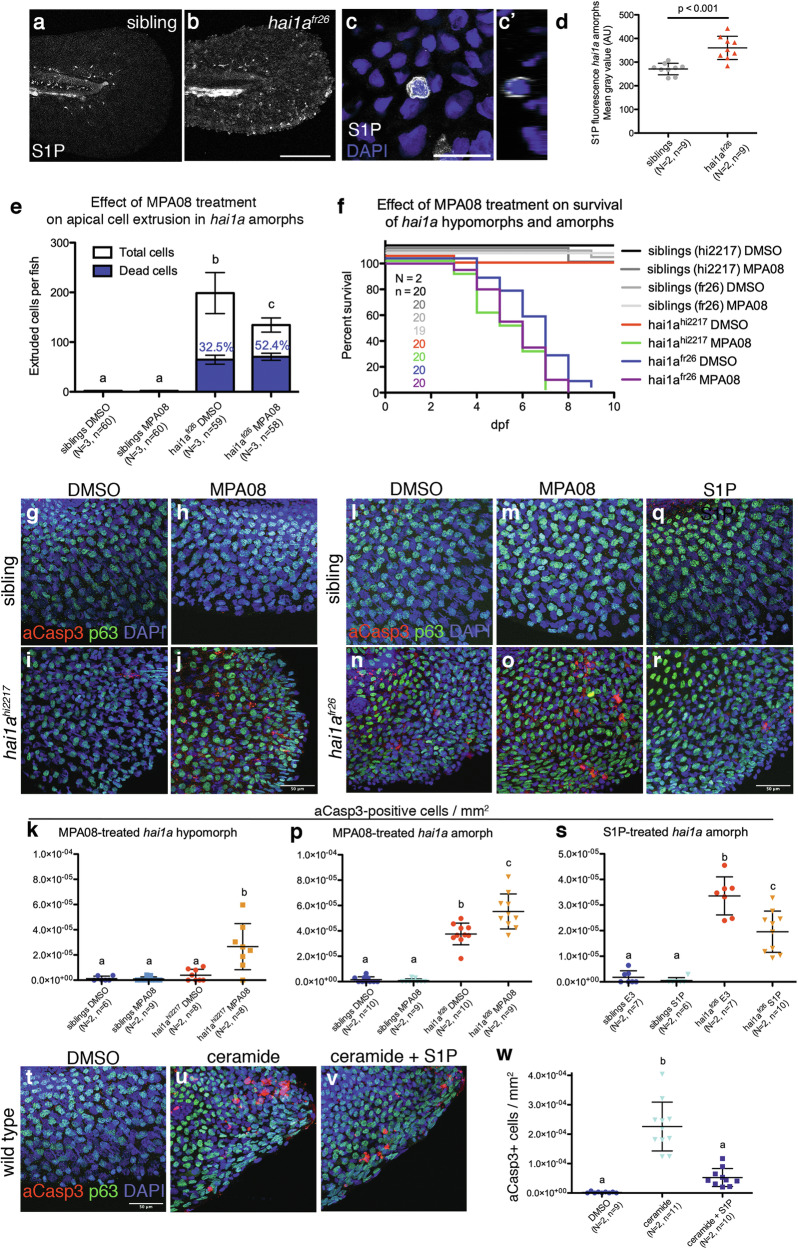
Sphingosine kinase activity modulates cell death in *hai1a*^*fr26*^ mutant epidermis. **a**–**d** S1P levels are elevated in *hai1a*^*fr26*^ mutants at 4 dpf. **a**–**c** Whole mount immunostaining in the caudal fin fold, anti-S1P (white), nuclei are labelled with DAPI (blue). **c** shows an individual extruding cell, with the orthogonal view shown in c’. **d** Quantification of S1P fluorescence in the caudal fins of embryos, normalised to fin area. For full dataset including SphK inhibition, see Fig. S3g. Scale bars: (**a**, **b**) 200 µm, (c) 20 µm. **e** Quantification of the numbers of extruded cells collected per fish at 4 dpf upon inhibition of sphingosine kinase by MPA08 treatment. White bars show the numbers of live cells, blue bars show the numbers of dead cells, with the proportion of dead cells indicated with blue text. **f** Survival curves showing the effect of sphingosine kinase inhibition on embryo viability. **g**–**j** Apoptotic cells in the caudal fin fold of 4 dpf *hai1a*^*hi2217*^ mutant embryos, with and without MPA08 treatment. Apoptotic cells are labelled with aCasp3 (red), basal keratinocytes with p63 (green), and nuclei using DAPI (blue). Scale bar = 50 μm. **l**–**o** Apoptotic cells in the caudal fin fold of 4 dpf *hai1a*^*fr26*^ mutant embryos, with and without MPA08 treatment. Apoptotic cells are labelled with aCasp3 (red), basal keratinocytes with p63 (green), and nuclei using DAPI (blue). Scale bar = 50 μm. **q**, **r** Apoptotic cells in the caudal fin fold of 4 dpf *hai1a*^*fr26*^ mutant embryos upon S1P treatment. Apoptotic cells are labelled with aCasp3 (red), basal keratinocytes with p63 (green), and nuclei using DAPI (blue). Scale bar = 50 μm. **k**, **p**, **s** Quantification of numbers of aCasp3-positive cells in the tail fins of embryos, normalised to fin area. **t**–**v** Apoptotic cells in the caudal fin fold of 4 dpf wild-type embryos, treated with ceramide to induce apoptosis (**u**), or concomitant treatment with ceramide and S1P (**v**). Apoptotic cells are labelled with aCasp3 (red), basal keratinocytes with p63 (green), and nuclei using DAPI (blue). Scale bar = 50 μm. **w** Quantification of numbers of aCasp3-positive cells in the tail fins of embryos, normalised to fin area. For (**d**), means of controls and mutants were compared using an unpaired, two-tailed Student’s *t*-test. For all other graphs, means were compared using one-way ANOVA with post-hoc Tukey’s multiple comparison test. *N* = number of biological replicates, *n* = number of fish per condition.

But which alternative pathways triggering cell death might be involved? In light of the requirement of Matriptase-dependent S1P signalling to promote tumour-suppressive ACE in both the hypomorphic *hai1a*^*hi2217*^ [29] and the amorphic *hai1a*^*fr26*^ (Fig. 3e) allele, and in light of its known effect promoting cell survival in multiple other contexts [21, 25], we tested whether S1P might also be involved in regulating the observed caspase-dependent epidermal cell death. Indeed, S1P levels, as assessed by anti-S1P whole mount immunostaining [35, 36], are significantly increased in amorphic *hai1a*^*fr26*^ mutants at 4 dpf compared to sibling controls, mainly in extruding peridermal cells (Fig. 3a–d and Fig. S3a-g; see Discussion). For temporal control of SphK activity and to circumvent earlier developmental defects of existing *sphk* mutants [37–39], we treated embryos with two independent pharmacological SphK inhibitors, MPA08 or SKI-II. These inhibitors significantly reduce S1P levels (Fig. S3a–g), and also increase the number of aCasp3-positive epidermal cells both in hypomorphic *hai1a*^*hi2217*^ (Fig. 3g–k and Fig. S3h–l) and amorphic *hai1a*^*fr26*^ (Fig. 3l-p and Fig. S3m–q) mutants compared to untreated mutant controls, coinciding with increased lethality of the mutants (Fig. 3f). In contrast, treatment of amorphic *hai1a*^*fr26*^ mutants with S1P, which is part of a formerly described pharmacological treatment reducing *hai1a*^*fr26*^ lethality [29], leads to a significant reduction in the number of aCasp3-positive epidermal cells (Fig. 3q–s). This indicates that S1P, in addition to promoting ACE, also reduces apoptotic cell death in the epidermis of *hai1a* mutant zebrafish, while levels of epidermal cell death strictly correlate with embryonic lethality.

S1P is known to elicit its pro-survival effect by counteracting the pro-apoptotic effect of ceramide, its opponent within the sphingolipid rheostat [21–25]. Indeed, upon treatment of wild-type embryos, water-soluble and cell-permeable C_2_ ceramide, which has been used as a pro-apoptotic agent in zebrafish embryos before [40], robustly induces apoptosis of epidermal cells at 4 dpf, while this effect is alleviated upon co-treatment with S1P (Fig. 3t–w). Treatment with (less, but still sufficiently water-soluble) longer-chain C_8_ ceramide, which has formerly been shown to induce apoptosis in multiple other contexts [41] or with the ceramide precursor, sphinganine, gives a similar pro-apoptotic effect on embryonic zebrafish keratinocytes (Fig. S3r–v). Together, this indicates that also in the embryonic zebrafish epidermis, S1P promotes cell survival by antagonizing the pro-apoptotic effect of ceramides, suggesting that in 4 dpf *hai1a*^*fr26*^ mutants, epidermal cell death might be due to imbalances within the endogenous sphingolipid rheostat.

### Pro-apoptotic ceramide levels are elevated in hai1a^fr26^ mutants at 4 dpf

**Fig. 4:**
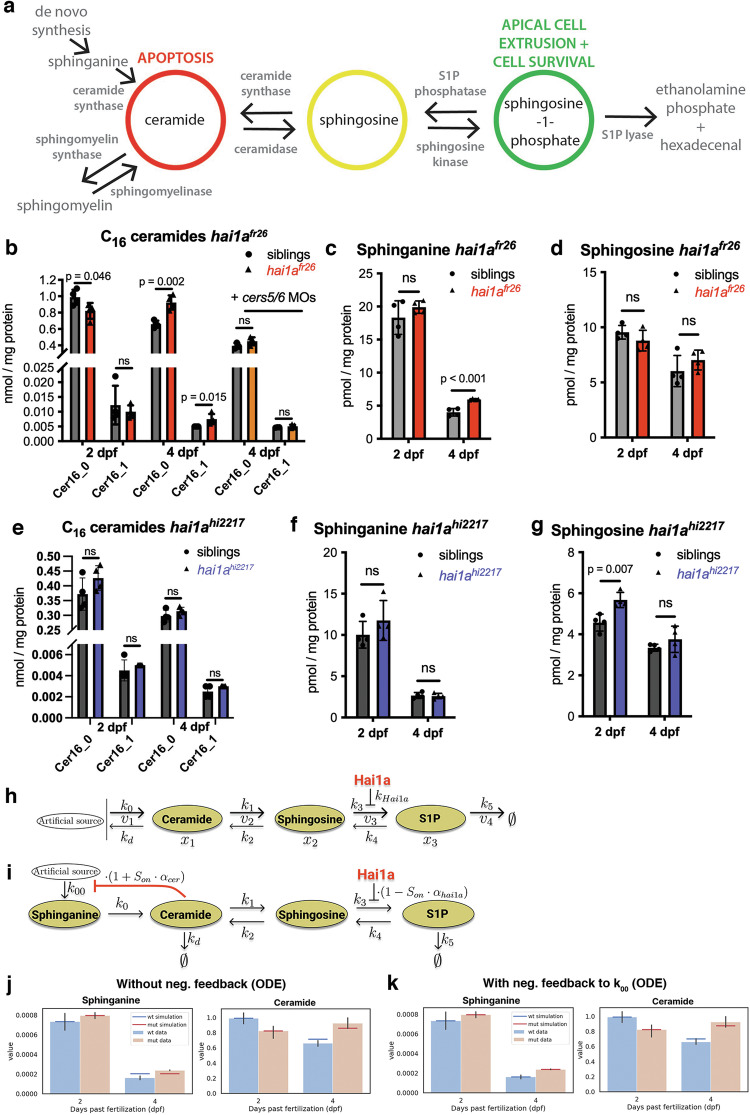
Sphingolipid levels in the *hai1a*^*fr26*^ mutant epidermis agree with mathematical modelling of the rheostat including a negative feedback loop controlling de novo ceramide synthesis. **a** Schematic of the sphingolipid rheostat showing the three principal lipids, ceramide, sphingosine, and S1P, and the enzymes responsible for their inter-conversion and de novo synthesis. **b**–**d** Lipidomic analysis of sphingolipids of the rheostat in *hai1a*^*fr26*^ amorph mutants and sibling controls. **b** Selected ceramide species (Cer_16_0_ and Cer_16_1_) at 2 dpf, 4 dpf, and 4 dpf upon MO-mediated knockdown of *cers5* and *cers6*. **c** Sphinganine levels at 2 dpf and 4 dpf. **d** Sphingosine levels at 2 dpf and 4 dpf. Means of controls and mutants within each condition were compared using an unpaired, two-tailed Student’s *t*-test. **e**–**g** Lipidomic analysis of sphingolipids of the rheostat in *hai1a*^*hi2217*^ hypomorph mutants and sibling controls, at 2 dpf and 4 dpf. Means of controls and mutants within each condition were compared using an unpaired, two-tailed Student’s *t*-test. **h** Simplified scheme of the sphingolipid rheostat model for MCA. Ceramide (${{\rm{x}}}_{1}$), sphingosine (${{\rm{x}}}_{2}$) and S1P (${{\rm{x}}}_{3}$) are considered as variables, reactions ${{\rm{v}}}_{1}-{{\rm{v}}}_{3}$ are reversible, S1P degradation (${{\rm{v}}}_{4}$) is considered irreversible. The system has an effective downstream flux (from ${{\rm{x}}}_{1}$ to ${{\rm{x}}}_{3}$) in the steady state. Ceramide synthesis (${{\rm{k}}}_{0}$) includes de novo synthesis and synthesis via sphingomyelin conversion and is modelled as a boundary condition, i.e. a constant synthesis rate of order zero is assumed. **i** Schematic of the ODE modelling, including sphinganine. A Boolean operator ${{\rm{S}}}_{{\rm{on}}}$ is active ${({\rm{S}}}_{{\rm{on}}}=1)$ in wild type and inactive ${({\rm{S}}}_{{\rm{on}}}=0)$ in the mutant, and S1P synthesis by *k*_*3*_ is described by ${{\rm{k}}}_{3,{\rm{app}}}={{\rm{k}}}_{3}\cdot \left(1-{{{\rm{S}}}_{{\rm{on}}}\cdot {\rm{\alpha }}}_{{\rm{hai}}1{\rm{a}}}\right),\,0.5\le {{\rm{\alpha }}}_{{\rm{hai}}1{\rm{a}}} < 1$ so that it is at least 50% lower in wild type than in the mutant. A possible negative feedback loop is shown, which is turned off when the ceramide concentration decreases below a certain threshold, and implemented via a scaling factor such that *k*_*00*_ can be described by ${{\rm{k}}}_{00,{\rm{app}}}={{\rm{k}}}_{00}\cdot {(1+{{\rm{S}}}_{{\rm{on}}}\cdot {\rm{\alpha }}}_{{\rm{cer}}}),\,-1 < {{\rm{\alpha }}}_{{\rm{cer}}} < 0$. **j** ODE model-predicted sphinganine and ceramide levels (horizontal lines) at 4 dpf compared to experimental lipidomics data (vertical bars) without the implementation of a negative feedback loop (${{\rm{\alpha }}}_{{\rm{cer}}}=0$). The 2 dpf data were set as the initial condition and are therefore perfectly met by the simulation results. **k** ODE model-predicted sphinganine and ceramide levels (horizontal lines) at 4 dpf compared to experimental lipidomics data (vertical bars) with the implementation of a negative feedback loop at the level of *k*_*00*_ (${{\rm{\alpha }}}_{{\rm{cer}}}=-1$).

Ceramides can be produced by multiple pathways, including de novo synthesis or salvage from sphingomyelin or sphingosine, while anti-apoptotic S1P can only be generated by a step-wise conversion of ceramides via sphingosine [21–25] (Fig. 4a). Therefore, we next studied whether sphingolipids of the rheostat are altered in *hai1a*^*fr26*^ mutants, with a particular focus on ceramides. In mammalian systems, only a subset of ceramide species is known to cause cell death, with the long-chain C_16_ ceramide being the best-studied pro-apoptotic ceramide species [42].

Lipidomic analysis revealed that at 2 dpf, before the appearance of apoptotic cells in the epidermis, levels of C_16_0_, C_20_0_, and C_22_1_ ceramides in *hai1a*^*fr26*^ mutants are significantly reduced compared to their siblings, whereas at 4 dpf, when apoptotic cells begin to appear, levels of C_16_0_, C_16_1_, C_24_0_, C_24_1_, and C_26_1_ ceramides are significantly increased (Fig. 4b and S4a, b). A similar time course was observed for sphingomyelins, which can be converted to ceramides by the action of sphingomyelinases (Fig. S4c, d), and for sphinganine, a precursor sphingolipid only found in the de novo synthesis of ceramide (Fig. 4c), while sphingosine, the intermediate lipid in the rheostat between ceramide and S1P, was unchanged at both time points (Fig. 4d).

Furthermore, hypomorphic *hai1a*^*hi2217*^ mutants, which do not display epidermal apoptosis, show no significant differences in C_16_ ceramide levels compared to controls at either time point (Fig. 4e), although there is a significant increase of potentially non-apoptotic ceramides similar to the increase in *hai1a*^*fr26*^ mutants (Fig. S4e, f). In addition, sphingomyelin (Fig. S4g, h) and sphinganine levels (Fig. 4f) of *hai1a*^*hi2217*^ mutants are unaltered compared to wild-type controls both at 2 and 4 dpf. Together, this suggests that C_16_ ceramides elevated in *hai1a*^*fr26*^ mutants at 4 dpf, but not at 2 dpf, are responsible for their progressive epidermal apoptosis.

### Mathematical modelling points to the presence of a negative feedback loop regulating ceramide synthesis

We have shown that *hai1a* mutants display increased Matriptase-1 activity, which in turn promotes the translocation of cytoplasmic eGFP-SphK1 (Fig. 4a) to its substrate at the plasma membrane [29]. Thus, *hai1a* mutants show increased levels of S1P (Fig. 3 and S3), whereas the SphK substrates on the other side of the rheostat, including ceramides, should be progressively depleted. The observed decrease of C_16_ ceramide levels at 2 dpf in *hai1a*^*fr26*^ amorphs compared to wild-type siblings and the weaker *hai1a*^*hi2217*^ mutants (Fig. 4b, e) are in line with these expected differences between wild type and mutant. However, the shift towards increased C_16_ ceramide levels in *hai1a*^*fr26*^ mutants at 4 dpf (Fig. 4b), together with increased levels of the ceramide de novo synthesis intermediate sphinganine (Fig. 4c), is puzzling. They point to either a second site of Matriptase influence on the sphingolipid rheostat, or to the existence of a negative feedback loop mediating long-term effects of the Matriptase—SphK axis on components of the de novo ceramide synthesis pathway.

To test the latter notion, we first applied Metabolic Control Analysis (MCA) [43, 44] to a simplified model of the core sphingolipid rheostat, including only ceramide, sphingosine, and S1P (Fig. 4h; see Supplementary Notes 1 for details). Hai1a and associated Matriptase activity were modelled via SphK as the sole entry point, affecting the S1P synthesis rate *k*_*3*_. MCA quantifies relative changes in the rheostat lipid concentrations upon small flux changes. In particular, the concentration control coefficients indicate changes in the steady-state concentrations upon changes in the reaction rates, and are used to compare the mutant with an increased S1P synthesis (*k*_*3*_) to the wild-type condition. The negative ceramide control coefficient of our MCA analysis indicates a decrease in ceramide concentration in the mutant compared to the wild type (Supplementary Notes 1). This result shows that the ceramide over-production observed in the *hai1a*^*fr26*^ mutants 4 dpf is not consistent with the reaction network structure of the rheostat as illustrated in Fig. 4h.

To confirm the MCA results and to determine whether a negative feedback loop could explain our experimental findings, we calibrated the rheostat model to the experimental data. To take all available data into account, we extended the model to also include sphinganine (Fig. 4i; see Supplementary Notes 2 for details). Ordinary differential equation (ODE) simulations of the sphingolipid rheostat model were used to test multiple combinations of possible *k* values, with the likelihood function (*L*(*θ*, *σ*)) describing how likely it is that the experimental results have been generated by the model for a particular combination of parameters *θ* and noise *σ* (see Methods). Without negative feedback (*α*_*cer*_ = 0), simulations confirm the MCA results, as the sphinganine and the C_16_ ceramide levels predicted by the model simulation do not agree with the experimental data even with the maximum likelihood estimate (Fig. 4j). Predicted C_16_ ceramide levels of 4 dpf *hai1a* mutants are slightly increased compared to 2 dpf mutants, consistent with the observed experimental shift; however, predictions fall outside the 95% confidence interval. Taken together, our model simulations yield ceramide values inconsistent with the experimental 4 dpf C_16_ ceramide data.

Next we implemented a negative feedback loop from ceramide to ceramide de novo synthesis and recalibrated the model, also taking into account the experimentally observed over-production of the ceramide precursor sphinganine in 4 dpf *hai1a* mutants (Fig. 4c). The feedback senses ceramide levels and promotes de novo synthesis of sphinganine (and thereafter ceramide) when ceramide concentrations drop below a certain threshold at 2 dpf (*α*_*cer*_ = −1, Fig. 4i). Indeed, the model predictions for sphinganine and ceramide including this negative feedback agree with the experimental lipid data at 4 dpf, as mutant and wild type model predictions are within the 95% confidence intervals of the experimental data (Fig. 4k).

In conclusion, our modelling approach suggests the presence of a negative feedback from ceramide levels to ceramide de novo synthesis, which can explain the experimentally observed over-production of ceramide at 4 dpf in the *hai1a*^*fr26*^ mutant. Matriptase-1 activity directly increases S1P levels by synthesis via ceramide, and with negative feedback from ceramide to its de novo synthesis, this initial ceramide decrease can lead to increased levels in the long run.

### Manipulation of the sphingolipid rheostat towards S1P alleviates cell death in hai1a mutant epidermis and towards ceramides enhances it

**Fig. 5:**
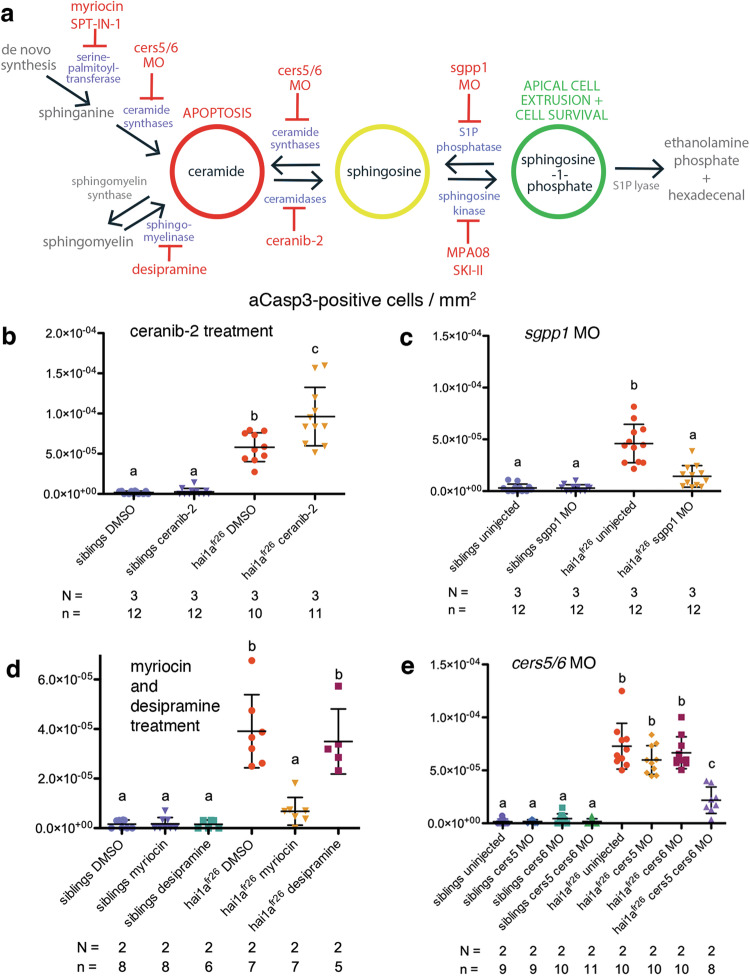
Manipulation of the sphingolipid rheostat enzymes or ceramide de novo synthesis alters cell death in the *hai1a*^*fr26*^ mutant. **a** Schematic of sphingolipid rheostat showing inhibition of ceramidases with ceranib-2, inhibition of sphingosine kinases with MPA08 or SKI-II, inhibition of S1P phosphatase with a MO targeting *sgpp1*, inhibition of serine palmitoyltransferase with myriocin or SPT-IN-1 and sphingomyelinases with desipramine, and MOs targeting *cers5* and *cers6* at the level of de novo synthesis and salvage from sphingosine. **b** Quantification of aCasp3-positive cells in the tail fins of 4 dpf embryos upon DMSO or ceranib-2 treatment, normalised to fin area. **c** Quantification of aCasp3-positive cells in the tail fins of control or *sgpp1* MO-injected 4 dpf embryos, normalised to fin area. **d** Quantification of aCasp3-positive cells in the tail fins of 4 dpf embryos upon DMSO, myriocin, or desipramine treatment, normalised to fin area. **e** Quantification of aCasp3-positive cells in the tail fins of control or *cers5* alone, *cers6* alone, or both *cers5* and *cers6* MO-injected 4 dpf embryos, normalised to fin area. For all graphs, means were compared using one-way ANOVA with post-hoc Tukey’s multiple comparison test. *N* = number of biological replicates, *n* = number of fish per condition.

To validate the actual in vivo involvement of the sphingolipid rheostat and its replenishment via ceramide de novo synthesis during the development of epidermal *hai1a* pathology, as assumed and predicted in our modelling, we tested if cell death rates in 4 dpf *hai1a*^*fr26*^ mutants can be altered by manipulating the rheostat, interfering with catalytic steps of ceramide—S1P conversion in either direction: pharmacologically blocking ceramidases, which usually tune the rheostat towards S1P, or applying MO technology to knock down the S1P phosphatase encoded by the *sgpp1* gene, which usually tunes the rheostat towards ceramides (Fig. 5a). In addition, to confirm the source of pro-apoptotic ceramides, we pharmacologically blocked all ceramide de novo synthesis, or its production from sphingomyelin (Fig. 5a). Finally, we blocked the specific production of pro-apoptotic C_16_ ceramide via salvage from sphingosine as well as its de novo synthesis from sphinganine, knocking down the ceramide synthase genes *cers5* and *cers6* [42, 45] (Fig. 5a).

Indeed, *hai1a*^*fr26*^ mutants treated from 3 to 4 dpf with the small molecule ceranib-2 to inhibit ceramidases display an even further increase in the number of dying epidermal cells compared to untreated mutants, while wild type siblings are unaffected (Fig. 5b and S5a–d). In contrast, 4 dpf *hai1a*^*fr26*^ mutants with concomitant knockdown of *sgpp1* display a significant alleviation of epidermal cell death rates (Fig. 5c and S5e-h). Treatment with two independent pharmacological inhibitors of serine palmitoyltransferase, myriocin or SPT-IN-1, to inhibit the de novo ceramide synthesis pathway, leading to reduced endogenous ceramide levels (Fig. S5q), also alleviates cell death rates, while treatment with desipramine to inhibit sphingomyelinases does not (Fig. 5d and S5i-r), confirming that de novo synthesis is necessary for the 4 dpf increase in apoptosis rates. Finally, 4 dpf *hai1a*^*fr26*^ mutants with concomitant knockdown of both *cers5* and *cers6* show significantly reduced epidermal cell death rates (Fig. 5e and S5s–z), coinciding with a normalization of C_16_ ceramide levels (Fig. 4b).

Together, this shows that epidermal cell death in 4 dpf *hai1a*^*fr26*^ mutants is caused by an increased flux of the sphingolipid rheostat towards replenishing ceramides via de novo synthesis.

### Early inhibition of sphingolipid de novo synthesis in wild-type embryos recapitulates S1P- and ceramide-related aspects of the later hai1a^fr26^ phenotype

**Fig. 6:**
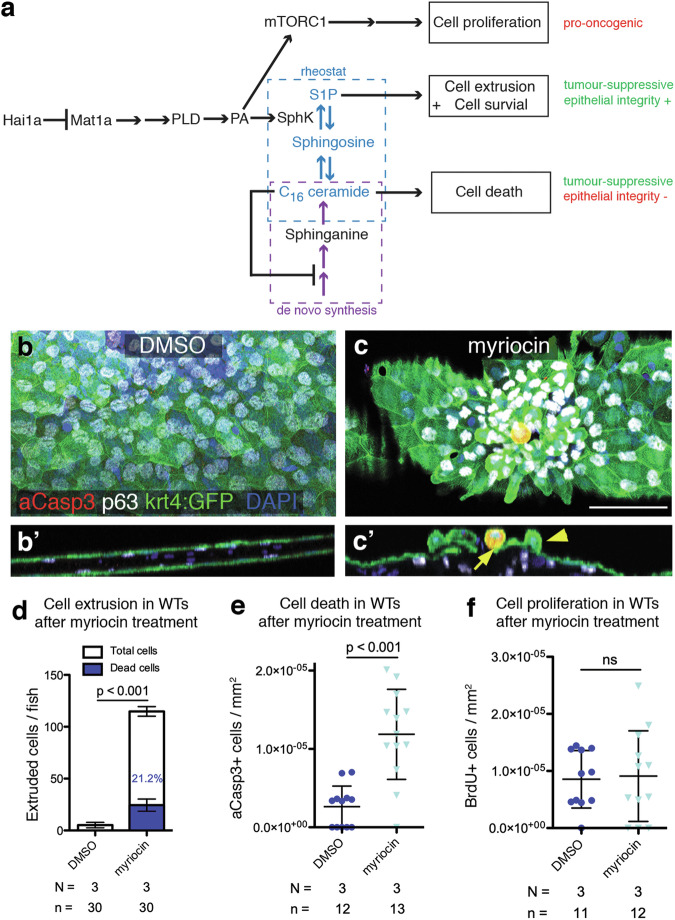
Early inhibition of ceramide synthesis in wild-type embryos recapitulates ACE and apoptosis phenotype. **a** Loss of Matriptase inhibition leads to parallel pro-oncogenic and tumour-suppressive pathways. Sphingolipid rheostat-mediated tumour-suppressive methods include S1P-dependent apical cell extrusion, which preserves epithelial integrity, and C_16_ ceramide-dependent apoptotic cell death, which may lead to loss of epithelial barrier function. When levels of C_16_ ceramide are above a certain threshold, a negative feedback loop prevents further de novo ceramide synthesis. In *hai1a*^*fr26*^ mutants by 2 dpf, ceramide levels drop due to sustained SphK activity catalysing S1P production, repression of de novo synthesis is lost, and resultant C_16_ ceramide levels trigger apoptosis. **b**-**c** Epidermal aggregates and apoptotic peridermal cells in the caudal fin fold of 4 dpf WT fish, transiently treated with inhibitor of de novo ceramide synthesis myriocin at 2 dpf, with orthogonal views (**b**’, **c**’) showing extruding live (yellow arrowhead) or apoptotic (yellow arrow) peridermal cells. Apoptotic cells are labelled with aCasp3 (red), peridermal cells with krt4:GFP (green), basal keratinocytes with p63 (white), and nuclei using DAPI (blue). Scale bar = 50 μm. **d** Quantification of the numbers of extruded cells collected per fish at 4 dpf upon inhibition of de novo sphingolipid synthesis by myriocin treatment at 2 dpf. White bars show the numbers of live cells, blue bars show the numbers of dead cells, with the proportion of dead cells indicated with blue text. **e** Quantification of aCasp3-positive cells in the tail fins of 4 dpf embryos upon myriocin treatment, normalised to fin area. **f** Quantification of proliferating cells in the tail fins of 4 dpf embryos upon myriocin treatment, normalised to fin area. For all graphs, means were compared via two-tailed, unpaired Student’s *t*-test. *N* = number of biological replicates, *n* = number of fish per condition.

To provide further functional in vivo evidence that the later death of keratinocytes observed in 4 dpf *hai1a*^*fr26*^ mutant embryos is indeed caused by the observed earlier reduction in ceramide levels at 2 dpf, and the resulting de-repression of the postulated negative feedback loop to ceramide de novo synthesis (Fig. 6a), we investigated whether the later phenotypic traits of *hai1a*^*fr26*^ mutants can be reproduced by depleting sphingolipids at an early stage in wild-type embryos. For this purpose, we transiently treated wild-type embryos at 2 dpf with the inhibitors of de novo ceramide synthesis, myriocin or SPT-IN-1, and examined later effects at 4 dpf on epidermal cell morphology, ACE, cell death, and proliferation.

In agreement with our model, early transient depletion of sphingolipid production in wild-type embryos results in the formation of epidermal aggregates and a significant increase in apical cell extrusion, including live and aCasp3-positive cells, as well as the total number of aCasp3-positive epidermal cells (Fig. 6b–e and S6a–d). However, of note, aspects of the *hai1a* phenotype unrelated to the sphingolipid rheostat, such as epidermal proliferation rates, are not affected (Fig. 6f).

In addition, to directly demonstrate the impact of the sphingolipid rheostat and its flow towards S1P on the negative feedback loop, we co-treated embryos with low (and per se sub-phenotypic amounts) of a *hai1a* MO to moderately increase Matriptase activity and thereby SphK activity. This leads to even further increased apical cell extrusion and apoptotic cell numbers in inhibitor-treated morphants (Fig. S6e–i) compared to embryos solely treated with the ceramide de novo synthesis inhibitors, thus displaying a clear synergistic effect. Together, these data suggest that the premature death of extruding keratinocytes in 4 dpf *hai1a*^*fr26*^ mutants is indeed the result of the SphK-driven increased flux within the sphingolipid rheostat towards S1P, the temporary depletion of ceramides and their over-compensatory replenishment via the de-repressed de novo synthesis pathway.

### Ceramide-dependent peridermal cell death compromises epidermal integrity and barrier function and thereby, the viability of hai1a^fr26^ mutants

Potentiating apoptosis in different cancer cell types, ceramides are well-known tumour suppressors [30]. Similarly, driving ACE, S1P has been shown to elicit tumour-suppressive effects even in the embryonic zebrafish epidermis [15], and in the context of *hai1a* mutations [28, 29]. In this light, amorphic *hai1a*^*fr26*^ mutants with the combined elevation of S1P and pro-apoptotic ceramides should have even higher tumour-suppressive capacities than hypomorphic *hai1a*^*hi2217*^ mutants; yet the amorphs die, whereas the hypomorphs heal and survive. Therefore, we wondered how the elevation of ceramide-driven cell death might affect the epidermis to compromise the viability of *hai1a*^*fr26*^ mutants, and why this is not so upon S1P-driven ACE.

**Fig. 7:**
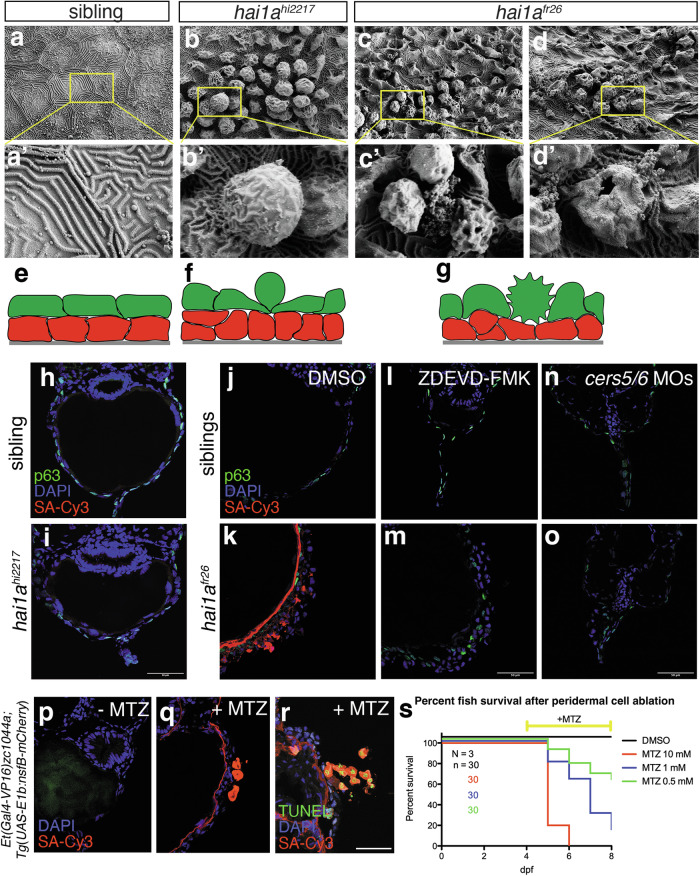
Embryo death is caused by apoptosis-induced loss of epidermal barrier. **a**–**d** Scanning electron microscopy images of the epidermal surface of sibling control (**a**), *hai1a*^*hi2217*^ hypomorphs (**b**), and *hai1a*^*fr26*^ amorph mutants (**c**, **d**). **e**–**g** Cartoons of the effects of *hai1a* mutation on epidermal morphology (basal keratinocytes in red, peridermal cells in green, underlying basement membrane in grey). **h**, **i** Biotin epidermal barrier assay on transverse cryosections in 4 dpf sibling control (**h**) and *hai1a*^*hi2217*^ (**i**) mutants. Basal keratinocytes are labelled with p63 (green), nuclei using DAPI (blue), and biotin using streptavidin-Cy3 (SA-Cy3, red). Scale bar = 50 μm. **j**, **k** Biotin epidermal barrier assay on transverse cryosections in 4 dpf sibling control (**j**) and *hai1a*^*fr26*^ (**k**) mutants. Basal keratinocytes are labelled with p63 (green), nuclei using DAPI (blue), and biotin using streptavidin-Cy3 (SA-Cy3, red). Scale bar = 50 μm. **l**–**o** Biotin epidermal barrier assay on transverse cryosections in 4 dpf sibling control (**l**, **n**) and *hai1a*^*fr26*^ (**m**, **o**) mutants, treated with caspase inhibitor ZDEVD-FMK (**l**, **m**) or injected with *cers5* and *cers6* MOs (**n**, **o**). Basal keratinocytes are labelled with p63 (green), nuclei using DAPI (blue), and biotin using streptavidin-Cy3 (SA-Cy3, red). Scale bar = 50 μm. **p**–**r** Biotin epidermal barrier assay upon peridermal cell ablation, without (**p**) or with (**q**) the prodrug metronidazole (MTZ), and using TUNEL to label dying cells (**r**). Nuclei are labelled using DAPI (blue), biotin using streptavidin-Cy3 (SA-Cy3, red), and dying cells using TUNEL-fluorescein (green). Scale bar = 50 μm. **s** Survival curves of control (DMSO) or MTZ-treated fish at different concentrations, with treatments starting from 4 dpf.

To gain first insights, we examined the architecture of the epidermal surface of *hai1a*^*hi2217*^ and *hai1a*^*fr26*^ mutants, using scanning electron microscopy (SEM). In 4 dpf control embryos, peridermal cells characterized by their apical microridges display the typical hexagonal shape and are tightly sealed to each other (Fig. 7a, e). In contrast, surface cells in either mutant have rounded into balloon-like shapes, as is typical for ACE. However, in *hai1a*^*hi2217*^ mutants (which lack epidermal apoptosis), extruding cells have retained their integrity, their microridges and proper cell-cell attachments (Fig. 7b, f). In contrast, in *hai1a*^*fr26*^ mutants, cells appear less regular and shrunken, often losing their microridges (Fig. 7c, d, g). In addition, their apical cell membrane occasionally appears ruptured (Fig. 7d), a feature described for cells undergoing secondary necrosis [46].

To investigate whether these morphological defects compromise the epidermal barrier, we performed an established penetration assay [47], testing whether the reactive component Sulfo-NHS-Biotin can pass the periderm of the epidermis to covalently bind to and thereby tag extracellular proteins inside the embryo, subsequently visualised by labelling with fluorescent streptavidin. In 4 dpf controls (*n* = 6/6) and *hai1a*^*hi2217*^ mutants (*n* = 6/6), the component was unable to penetrate the epidermis, and neither epidermal keratinocytes themselves, nor interior extracellular structures like the skin basement membrane are labelled (Fig. 7h, i). In contrast, amorphic *hai1a*^*fr26*^ mutants show streptavidin labelling throughout all layers of the epidermis and the underlying basement membrane (*n* = 6/6; Fig. 7j, k), while inhibition of caspase-3 activity with ZDEVD-FMK (*n* = 8/8; Fig. 7l, m) as well as MO-mediated knockdown of ceramide synthases 5 and 6 in mutant embryos (*n* = 6/6; Fig. 7n, o) abolishes this labelling. Together, this indicates that apoptosis, but not apical extrusion, of peridermal cells compromises the epidermal integrity.

Finally, to test whether such loss of peridermal integrity might actually be the cause of *hai1a*^*fr26*^ mutant lethality, we specifically ablated peridermal cells in wild-type embryos, using the well-established nitroreductase / metronidazole (MTZ) approach that allows temporally controlled cell ablation [48]. Indeed, upon treatment with the prodrug MTZ, embryos carrying both the Tg(UAS-E1b:nfsB-mCherry) effector transgene and the periderm-specific Et(Gal4-VP16)^zc1044a^ driver transgene, display peridermal cell death (Fig. 7r), accompanied by loss of epidermal barrier function (Fig. 7p–r). In addition, embryos begin to die within 8 h after MTZ application and in an MTZ concentration-dependent manner (Fig. 7s). Together, this strongly suggests that in *hai1a*^*fr26*^ mutants, it is indeed the ceramide-induced apoptosis of peridermal cells and the resulting loss of epidermal integrity that ultimately causes the death of the animal.

## Discussion

We previously described S1P production downstream of Matriptase signalling as a result of increased PLD-mediated phosphatidic acid (PA) production recruiting sphingosine kinase (SphK) to the plasma membrane, where S1P promotes ACE of live pre-neoplastic keratinocytes, counteracting the parallel hyperplastic effect of elevated Matriptase signalling and eventually leading to perfect healing of hypomorphic zebrafish *hai1a*^*hi2217*^ mutants [29]. Now, while investigating the basis of the lethality of amorphic *hai1a*^*fr26*^ mutants, we identified a third effect of Matriptase, inducing keratinocyte apoptosis (Fig. 6a). This effect only becomes obvious when Matriptase activity is particularly high (upon complete loss of its cognate Hai1a inhibitor in amorphs, but not upon partial loss of Hai1a activity in hypomorphs) and is caspase-dependent, yet tp53-independent. Of note, keratinocyte apoptosis in *hai1a*^*fr26*^ amorphs can be alleviated upon exogenous S1P application, and induced in *hai1a*^*hi2217*^ hypomorphs upon inhibition of endogenous S1P production via sphingosine kinase, pointing to a pro-survival effect of S1P that occurs in combination with its ACE-promoting effect (Fig. 3). Consistently, we found S1P levels to be particularly up-regulated in extruding keratinocytes of *hai1a*^*fr26*^ amorphs (Fig. 3c). This is remarkable, as Matriptase-1 should be rather homogeneously up-regulated throughout the tissue, possibly pointing to the existence of some Notch-dependent lateral inhibition mechanisms (JA and MH, unpublished data). Via these mechanisms, single cells within the epithelium could be sorted out that display unrestricted S1P production, thereby committing these particular cells for ACE, consistent with the single cell synthesis of S1P formerly reported in epithelial cell culture systems [49]. In addition, we present evidence that the death of keratinocytes in *hai1a*^*fr26*^ amorphs is ceramide-dependent and due to imbalances in the endogenous ceramide-S1P sphingolipid rheostat. Thus, levels of pro-apoptotic C_16_ ceramides are significantly up-regulated in 4 dpf amorphic *hai1a*^*fr26*^ mutants, coinciding with the onset of epidermal cell death (Fig. 4). In addition, exogenous application of ceramides and pharmacological interference tuning the endogenous sphingolipid rheostat towards ceramide promote epidermal cell death rates, while genetic interference tuning the rheostat towards S1P alleviates them (Figs. 3 and 5).

Previous studies have shown increased SphK activity and S1P levels downstream of oncogenic KRAS signalling in HEK293T cells, leading to a decrease in C_16_ ceramide [50]. This decrease is similar to what we have found for 2 dpf *hai1a*^*fr26*^ mutants due to oncogenic Matriptase signalling (Fig. 4). However, we also found that such shifts in the sphingolipid rheostat can be reverted over time, and that these fluctuations lead to different cell fate outcomes, despite ongoing oncogenic signalling. Via mathematical modelling we demonstrate that the shift from reduced ceramide levels at 2 dpf to increased levels at 4 dpf can be explained by a negative feedback loop. Over-compensation of this feedback loop leads to increased de novo ceramide synthesis once ceramide levels have dropped below a certain threshold (Figs. 4 and 6). This feedback to ceramide de novo synthesis is consistent with the experimentally observed concomitant rise in the levels of sphinganine, the direct ceramide precursor within the pathway (Fig. 4), and with the reported slow timescale of ceramide de novo synthesis (hours to days) [42]. Compensatory ceramide generation via the salvage pathway by sphingomyelinases is less likely, as it occurs in a time frame of minutes [42]. Additionally, we did not detect any decrease in sphingomyelin levels; rather, they generally reflected the levels of corresponding ceramide species (Fig. S4). This, combined with the fact that sphingomyelinase inhibition did not affect apoptotic cell numbers (Fig. 5d), indicates that ceramide is fuelling sphingomyelin production and thereby ceramide storage, and not vice versa. Together, our data are nicely in line with, and confirm in vivo, a recent report that in vitro ceramide analogues can bind to the serine palmitoyltransferase complex member ORMDL3 and inhibit the first, rate-limiting step of sphingolipid de novo synthesis [51].

Of note, the ceramide derivative and other central component of the sphingolipid rheostat, sphingosine, is also known to be cytotoxic, including in zebrafish [52]. Yet, we did not detect any significant difference in sphingosine levels in our lipidomics analyses of amorphic *hai1a*^*fr26*^ mutants, which nevertheless show increased apoptosis in the epidermis. On the other hand, we did detect significantly increased sphingosine levels in the hypomorphic *hai1a*^*hi2217*^ mutants at 2 dpf, which do not show increased epidermal apoptosis (Fig. 4d and g). Additionally, ceramidase inhibition (which should lead to decreased sphingosine levels) significantly increased apoptotic cell numbers (Fig. 5b). Together, this makes it unlikely that sphingosine is mediating epidermal cell death in *hai1a*^*fr26*^ mutants.

Although we have not determined the exact sub-cellular compartments affected by the altered sphingolipid rheostat, previous studies have shown that pro-apoptotic ceramide-producing enzymes CerS5 and CerS6 localise to mitochondrial-associated membranes (MAMs) [42], sites of physical interaction between the endoplasmic reticulum and mitochondria. Thus long-chain ceramides generated de novo in MAMs are rapidly transferred to the mitochondria, causing MOMP and apoptosis [53]. On the other hand, we hypothesize that cell-surface Matriptase activity induces an increase in S1P production in the plasma membrane. Therefore, it seems likely that the corresponding decrease in the plasma membrane of sphingosine and subsequently ceramides, which we detected at 2 dpf (Fig. 4) is sensed by the cell to induce de novo ceramide synthesis in the ER and MAMs.

Dysregulated Matriptase signalling is at the heart of numerous carcinomas in humans [54, 55]. Therefore, unravelling the multitude of downstream pathways initiated by this promiscuous protease is of great interest for cancer research and the development of effective cancer treatments. Very little is known about bioactive lipid signalling mediated by Matriptase activity. We have previously demonstrated the involvement of SphK and S1P in inducing tumour-suppressive ACE, now revealing a more general involvement of the entire sphingolipid rheostat and pro-apoptotic C_16_ ceramides (Fig. 6). We also discovered that very long-chain ceramides C_24_ and C_26_ are elevated in both *hai1a* mutant alleles (Fig. S4), independent of apoptosis, opening up scope for further research into the functions of these ceramides and their corresponding synthase enzymes as mediators of other Matriptase effects.

Our findings on the roles and interconnection of S1P and pro-apoptotic C_16_ ceramides in the pre-neoplastic zebrafish epidermis might also be relevant for carcinogenesis in general, and beyond the involvement of Matriptase-1. The promotion of cell death as a cancer treatment is the subject of intense research and multiple clinical trials. In particular, the sphingolipid pathway has become an attractive target due to the ability of ceramide to promote tumour-suppressive apoptosis [30, 38, 56, 57] and even to circumvent multi-drug resistance [38, 58]. Indeed, many existing chemotherapeutics and radiation therapies promote apoptosis via ceramide production [59]. Similarly, blocking S1P production using the SphK inhibitor Safingol enhances Fenretinide-induced, ceramide-mediated apoptosis, even in cancer cell lines lacking functional tp53 [58]. On the other hand, ceramide-induced apoptosis can have lethal side effects, such as the acute gastrointestinal syndrome caused by apoptosis in the endothelial cells of the small intestine [60]. In addition, high expression of CerS5 has been reported to predict a poor prognosis in gastric and colon cancer [31, 32].

This highlights a need to better understand the sphingolipid rheostat in order to modulate it in a manner which leads to tumour suppression and cell loss in cancer, without adversely affecting tissue function, or causing unwanted apoptosis in nearby normal tissues. One such example is the application of exogenous S1P to prevent radiation-induced ceramide-dependent apoptosis [60]. Our results obtained for zebrafish *hai1a* mutants are in line with, and shed further light onto, these double faces of both S1P and pro-apoptotic ceramides. The pro-survival effect of S1P, while pro-oncogenic and deleterious for organisms in multiple other contexts of carcinogenesis [25], is beneficial in the zebrafish epidermis when occurring in combination with tumour-suppressive ACE to ensure that extruding cells do not compromise the epidermal barrier. Along the same lines, ceramides, although tumour-suppressive and therefore beneficial for organisms in multiple other contexts of carcinogenesis, have detrimental and life-threatening effects, inducing apoptosis and secondary necrosis of epidermal cells even before they extrude, thereby severely compromising the epithelial barrier (Fig. 7). Of note, this is what we obtained when treating the zebrafish *hai1a* mutants with a pharmacological inhibitor of ceramidases (Fig. 5b and S5a–d), the same strategy as applied in human cancer treatment using the ceramidase inhibitor B13, which tunes the sphingolipid rheostat towards ceramide [61, 62]. In this light, such strategies should be taken cautiously and not applied for carcinomas of epithelial cell types lining the exterior or the lumen of internal organs and fulfilling crucial barrier functions (such as gastrointestinal, prostate or mammary gland carcinomas). Instead, in these cases, it might be more reasonable to apply strategies to tune the sphingolipid rheostat towards S1P (e.g. with inhibitors of S1P phosphatase; see Fig. 5c and S5e–h), thereby promoting ACE of neoplastic cells without compromising the epithelial barrier of the tissue. However, the untreated zebrafish *hai1a* mutants per se, which display the same shift due to increased activity of SphK, also teach us that even the effects of such treatments to tune the sphingolipid towards S1P can be highly dose-dependent. Thus, only *hai1a*^*hi2217*^ hypomorphs heal and survive, whereas *hai1a*^*fr26*^ amorphs, with an even stronger shift towards S1P, worsen over time and eventually die due to adverse side effects of feedback to de novo ceramide synthesis and the apoptotic death of epithelial cells. Yet, our findings also point to possible strategies to avoid such long-term replenishment of ceramides, targeting CerS5 and CerS6 ceramide synthases (see Fig. 5e and S5s–z).

## Methods

### Animal care

All zebrafish experiments were approved by the local and federal animal care committees (LANUV Nordrhein-Westfalen: 84-02.04.2012.A251, 84-02.04.2012.A390, 81-02.04.40.2022.VG005, 81-02.04.2018.A281, 81-02.04.2022.A104; City of Cologne: 8.87-50.10.31.08.129) and by the University of Cologne. Zebrafish embryos were raised according to standard protocols and were randomly assigned to treatment groups. No effort was made to separate embryos by sex, and blinding was not performed when assessing the outcome of experiments.

### Lines used in this study

The following zebrafish lines were used in this study: *hai1a*^*hi2217Tg*^ (ZDB-ALT-040924-4) [26]; *hai1a*^*fr26*^ (ZDB-ALT-200618-2) [29]; *tp53*^*zdf1*^ (ZDB-ALT-0504282) [63]; *Tg(UAS-E1b:nfsB-mCherry*) (ZDB-ALT-070316-1) [64]; *Et(Gal4-VP16)*^*zc1044a*^ (ZDB-ALT-120604-1) [64]; *Tg(krt4:GFP)*^*gz7*^(ZDB-ALT-080207-1) [65].

### Counting of apically extruded cells

Cells extruded from zebrafish embryos were collected at appropriate time points and stained with Trypan blue to distinguish live and dead cells, as previously described [29].

### Antibody staining

For whole mount immunostaining using most antibodies, embryos were fixed at appropriate time points in 4% PFA overnight at 4 °C, washed extensively in PBS-Triton X-100 (0.5%), and incubated in blocking buffer (4% foetal calf serum and 1% DMSO in PBS-Triton X-100 0.5%). Primary antibody staining was performed at 4 °C overnight, while secondary antibody staining was generally performed at room temperature for 1.5 h. DAPI was used at 1:1000 for 15 min in wash buffer to counterstain nuclei. For staining cryosections, the same protocol was performed, and sections were mounted in MOWIOL containing DAPI. The primary antibodies were diluted 1:250 in blocking buffer and were: rabbit polyclonal anti-cleaved caspase 3 (RRID:AB_397274, BD Biosciences, Heidelberg, Germany, Cat# 559565 or Cat#570524), mouse monoclonal anti-p63 (RRID:AB_10588476, Biocare Medical, Pacheco, CA, USA, Cat# CM 163 C), rabbit polyclonal anti-BrdU (RRID:AB_2813902, Abcam, Amsterdam, Netherlands, Cat# 152095), chicken polyclonal anti-GFP (RRID:AB_2534023, Invitrogen, Darmstadt, Germany, Cat#A10262). Secondary antibodies were diluted 1:500 and were all purchased from Thermo Fisher Scientific (Darmstadt, Germany): goat anti-rabbit Cy3 (RRID:AB_2534029, Cat# A10520), goat anti-chicken Alexa Fluor 488 (RRID:AB_2534096, Cat# A11039), goat anti-mouse Alexa Fluor 488 (RRID:AB_2534069, Cat# A11001), goat anti-mouse Alexa Fluor 647 (RRID:AB_2535804, Cat# A21235), goat anti-mouse Cy3 (RRID:AB_2534030, Cat# A10521).

For whole mount immunostaining of S1P, embryos were fixed in 4% PFA for 1 h at room temperature in glass vials, washed extensively in TBS, and permeabilised for 20 min in 0.5% Saponin in TBS. Following further TBS washes, embryos were incubated in blocking buffer (10% sheep serum in TBS) overnight at 4 °C. Primary antibody (mouse anti-S1P LT1002, Echelon Biosciences, Salt Lake City, Utah, USA, Cat# Z-P300) was diluted 1:50 in blocking buffer, and incubated for 2 h at room temperature. The primary antibody was removed and the embryos were washed extensively in 1% sheep serum in TBS, and incubation in the secondary antibody (goat anti-mouse Alexa Fluor 488, 1:250) was carried out for 1.5 h at room temperature. Following nuclear staining using DAPI, embryos were mounted and imaged as usual using a laser scanning confocal microscope.

### Small molecule treatments

All small molecule treatments were bath-applied in 1x E3 embryo medium (60x stock: 295 mM NaCl, 10 mM KCl, 20 mM CaCl_2_, 20 mM MgSO_4_) to zebrafish embryos in 24-well plates, with controls incubated in the respective vehicle. All small molecules were prepared as a stock in DMSO except Huzzah-S1P which was prepared in 1x E3 embryo medium. Final concentrations were as follows: ZDEVD-FMK 10 µM (BD Biosciences, Cat# 550378), MPA08 50 µM (Tocris, Wiesbaden, Germany, Cat# 5803), C_2_ ceramide 10 µM (Enzo Life Sciences, Lörrach, Germany, Cat# BML-SL100-0005), Huzzah-S1P 10 µl/ ml (Avanti Polar Lipids, Birmingham, AL, USA, Cat# 360492 P), C_8_ ceramide 117 µM (Avanti Polar Lipids, Cat# 860508 P; complexed 1:1 with fatty acid-free bovine serum albumin 1 mM, Carl Roth, Karlsruhe, Germany, Cat#0052.1), sphinganine 0.5 µg/ ml (Avanti Polar Lipids, Cat# 860498 P), ceranib-2 1.4 µM (Merck Cat# SML0607), myriocin 25 µM (Merck, Darmstadt, Germany, Cat# M1177), desipramine hydrochloride 7.5 µM (Merck Cat# D3900), SKI II 25 µM (Merck Cat# S5696), SPT-IN-1 3.4 µM (MedChemExpress, Sollentuna, Sweden, Cat# HY-125351). For myriocin or SPT-IN-1 treatment, two treatment paradigms were used: acute treatment of mutant embryos from 3–4 dpf to prevent apoptosis, or transient treatment of WT embryos overnight from 2–3 dpf followed by extensive washout at 3 dpf.

### Bromodeoxyuridine incorportation

Embryos were incubated at appropriate time points in 10 mM BrdU (Sigma-Aldrich, Taufkirchen, Germany, Cat# B5002-5G) dissolved in 1x E3 embryo medium for 3 h in 24-well plates at 28 °C. BrdU was then washed out with fresh E3 medium, and the embryos were incubated for a further 1 h at 28 °C, then fixed in 4% PFA at room temperature for 1 h. Following extensive washes in PBS-Triton 0.5%, antigen retrieval was performed using 2 N HCl at room temperature for 30 min. Embryos were washed extensively, followed by antibody staining as described above.

### TUNEL assay

Terminal dUTP nick-end labelling was performed using the In Situ Cell Death Detection Kit (Sigma-Aldrich, Cat# 11684817910) following the manufacturer’s instructions. Briefly, fixed embryos at various time points were incubated in enzyme labelling mix for 30 min at 37 °C, washed, and the fluorescein labelling was imaged immediately.

### Lipidomics analysis

Lipidomics analyses were performed at the CECAD Lipidomics/ Metabolomics core facility. Lipids from the yolk and from the brain were avoided by using only embryo tails. At 2 or 4 dpf, tails were truncated using a scalpel from 300 anaesthetized embryos per condition in quadruplicate, collected, and snap frozen at −80 °C.

Levels of selected sphingolipid species (ceramides, sphingomyelins, sphingosine, and sphinganine) were determined by Liquid Chromatography coupled to Electrospray Ionization Tandem Mass Spectrometry (LC-ESI-MS/MS) using previously described procedures [66–68] with several modifications. For lipid analysis, the embryo tail samples (in quadruplicate) were homogenized in 300 µl of Milli-Q water using the Precellys 24 Homogenisator (Peqlab, Erlangen, Germany) at 6,500 rpm for 30 sec. The protein content of the homogenate was routinely determined using bicinchoninic acid.

#### Ceramides and sphingomyelins

To 100 µl of homogenate 500 µl of methanol, 250 µl of chloroform and internal standards (127 pmol ceramide 12:0 and 124 pmol sphingomyelin 12:0, both Avanti Polar Lipids) were added. Lipid extraction, alkaline hydrolysis of glycerolipids and LC-ESI-MS/MS analysis of ceramides and sphingomyelins were performed as described [66, 67].

#### Sphingosine and sphinganine

The sphingoid bases sphingosine and sphinganine were extracted by methanol precipitation [60]: To 100 µl of homogenate 400 µl of methanol with 0.1% formic acid and internal standards (121 pmol sphingosine (d17:1) and 130 pmol sphinganine (d17:0), both Avanti Polar Lipids) were added, followed by shaking at 900 rpm/min in a ThermoMixer (Eppendorf, Wesseling, Germany) at 20 °C for 15 min. After centrifugation (16,100 x g, 15 min, 4 °C), the supernatant was transferred to a new tube and dried under a stream of nitrogen. The residues were resolved in 100 µl of methanol and sonicated for 5 min. After centrifugation (16,100 × g, 15 min, 4 °C), 90 µl of the clear supernatants were transferred to autoinjector vials.

Levels of the sphingoid bases sphingosine and sphinganine were determined by LC-ESI-MS/MS using a procedure previously described [60] with several modifications: 10 µl of sample were loaded onto a Core-Shell Kinetex C18 column (150 mm × 2.1 mm ID, 2.6 µm particle size, 100 Å pore size, Phenomenex, Aschaffenburg, Germay), and sphingosine and sphinganine were detected using a QTRAP 6500 triple quadrupole/linear ion trap mass spectrometer (SCIEX, Framingham, MA, USA). The LC (Nexera X2 UHPLC System, Shimadzu, Langenfeld, Germany) was operated at 30 °C and at a flow rate of 0.3 ml/min with a mobile phase of 0.1% formic acid in water (solvent A) and 0.1% formic acid in acetonitrile/tetrahydrofuran 50:50 (v/v) (solvent B). Sphingoid bases were eluted with the following gradient: initial, 42.5% B; 0.6 min, 42.5% B; 5 min, 100% B; 6 min, 100% B; 6.5 min, 42.5% B; 10 min 42.5% B. Sphingoid bases were monitored in the positive ion mode with their specific Multiple Reaction Monitoring (MRM) transitions (Supplementary Table 1). The instrument settings for nebulizer gas (Gas 1), turbogas (Gas 2), curtain gas, and collision gas were 30 psi, 30 psi, 40 psi, and medium, respectively. The Turbo V ESI source temperature was 400 °C, and the ionspray voltage was 5.5 kV. The values for declustering potential (DP), entrance potential (EP), collision energy (CE), and cell exit potential (CXP) of the different MRM transitions are listed in Supplementary Table 1.

The quantifier peaks of endogenous sphingosine (d18:1) and sphinganine (d18:0) and the internal standards sphingosine (d17:1) and sphinganine (d17:0) were integrated using the MultiQuant 3.0.2 software (SCIEX). Endogenous sphingoid bases were quantified by normalizing their peak areas to those of the respective internal standard, and the concentration calculated relative to that of the standard. The normalized concentrations were then normalized to the protein content of the cell homogenate.

### Morpholino oligonucleotide-mediated knockdown

All morpholinos were designed by and obtained from Gene Tools (Philomath, OR, USA), and 1 mM stocks in H_2_0 were maintained at room temperature. MOs were diluted in Danieau buffer and injected with Phenol red in a volume of 3 nl into fertilized eggs. MO sequences and dilutions were as follows: *p63* 5’ - CCCTAGTTTTCTTCCTTTTATCCCC- 3’, 1:50 (ZDB-MRPHLNO-050204-5) [34]; *cers5* 5’ - AGAGCGCAGCCATGTTTAGAAGAAA- 3’, 1:5 (this study); *cers6* 5’ - ACGCCAGAATGCCTGCCATTTCCAG- 3’, 1:5 (this study); *sgpp1* 5’ - CCTGCCATGTCCAGCGACAAAACTG- 3’, 1:10 (this study); *spint1a (hai1a)* 5’ - ACCCTGAGTAGAGCCAGAGTCATCC - 3’, 1:100 (ZDB-MRPHLNO-071218-5) [26].

### Cell ablation

To ablate peridermal cells, *Tg(UAS-E1b:nfsB-mCherry*); *Et(Gal4-VP16)*^*zc1044a*^ embryos (sorted for the GFP heart marker and for mCherry) were treated essentially as described [69]. Briefly, starting at 4 dpf larvae were incubated in either DMSO or 10 mM Metronidazole (Sigma-Aldrich) at 28 °C for 6–8 h, then fixed in 4% paraformaldehyde. For survival assays, larvae were incubated in either DMSO or Metronidazole at concentrations from 0.5 – 10 mM from 4 dpf, and the solution was changed daily.

### Epidermal barrier assay

The assay was performed essentially as described [47]. Briefly, EZ-Link Sulfo-NHS-Biotin (Thermo Fisher) was dissolved in at 1 mg/ml in PBS ( + MgCl2, +CaCl_2_; Sigma-Aldrich), and 1 ml was added to 10 embryos in 24 well flat-bottom plates, then incubated for 30 min at 4 °C. Embryos were washed in 100 mM glycine in PBS, then fixed in 4% PFA for 1 h at room temperature. Embryos were incubated in 30% sucrose and embedded in 1.5% agarose, 5% sucrose in PBS before cryosectioning. Finally, to visualise biotin, cryosections were incubated after antibody staining 15 min at RT in either Streptavidin-Cy3 from *Streptomyces avidini* (Sigma-Aldrich) diluted 1:100 in blocking buffer or Streptavidin, DyLight 488 (Vector Laboratories, Newark, CA, USA) at a concentration of 5 µg/ml. Sections were washed briefly twice in PBS-Triton X-100 (0.5%) and coverslips were mounted using MOWIOL-DAPI.

### Microscope image acquisition

Bright field images were acquired using a Leica (Wetzlar, Germany) M165FC stereo microscope with DFC425C camera and Leica Application Suite V3.8 software, after mounting embryos in 3% methyl cellulose/ 1x tricaine (Sigma-Aldrich). Fluorescent images were acquired using a Zeiss (Göttingen, Germany) LSM 710 confocal microscope and 10x/0.3 EC Plan NeoFluar, 20x/0.8 Plan-Apochromat, or 40x/1.1 W Korr LD C-Apochromat objectives and Zen 2.3 SP1 software. Images were further processed using Fiji/ Image J software including generation of mosaic images, orthogonal projections, maximum intensity projections, and adjustment of brightness and contrast. For quantification of anti-S1P staining mean grey value, sum of slices projection was used.

### Quantification and statistical analysis

In general, results are presented in figures as the mean with error bars representing standard deviation. The number of biological replicates (N) and the total number of embryos (n) is indicated in the figure or in the figure legend. For all lipidomics analyses, *N* = four biological replicates with *n* = 300 tails per replicate, except for *cers5/6* morphant siblings, where one biological replicate was excluded as an outlier. Statistical analysis was performed using GraphPad Prism (Boston, MA, USA) software, including unpaired, two-tailed Student’s *t*-test to compare two conditions, or one-way ANOVA with post-hoc Tukey’s multiple comparison test to compare multiple groups. No pre-specified effect size was used to ensure adequate power; sample size was estimated according to previous results. Data were tested for normal distribution and equal variance. Exact *p*-values for statistically significant differences are indicated in figures, while comparisons with *p* > 0.05 were considered as not significant and denoted ns. For ANOVA comparisons, different letters above a graph indicate a statistically significant difference.

### Mathematical modelling

#### Methods/implementation details

The ordinary differential equation system with mass action kinetics was implemented in the Systems Biology Markup Language (SBML) [70]. Together with the experimental data, a PEtab file [71] was created that contains (I) the SBML model of the sphingolipid rheostat, (II) an experimental conditions file with the *hai1a*^*fr26*^ mutant and wild-type specifications, (III) a parameter file for all rate constants of the model, (IV) an observables file which maps the species of the model together with an error model to experimental readouts for fitting and (V) a yaml file that maps these files together. The likelihood function *L*(*θ*, *σ*) with independent and additive normally distributed noise *σ*, *N* replicates and *T* different measurement time points is given by the product

$$L(\theta ,\sigma )={\prod }_{j=1}^{N}{\prod }_{k=1}^{T}\frac{1}{\sqrt{2\pi }\sigma }\exp \left(-\frac{{\left(y\left({t}_{k},\theta \right)-{m}_{j}\left({t}_{k}\right)\right)}^{{\rm{T}}}\left(y\left({t}_{k},\theta \right)-{m}_{j}\left({t}_{k}\right)\right)}{2{\sigma }^{2}}\right),$$

with the solution $y\left({t}_{k},\theta \right)$ of the ODE and the respective measured value ${m}_{j}\left({t}_{k}\right)$ at the time point ${t}_{k}$. One noise parameter $\sigma$ for all time points was estimated for each species individually. As the experimental readouts were measured in different units that could not be converted into each other, scaling factors for all species were introduced and estimated along with the kinetic and noise parameters. The toolbox pyPESTO [72] was used for model calibration. Parameter estimation was done with the hybrid Hessian approximation algorithm Fides [73] with 100 starts. Since measured values were only available at two time points, measurements at 2 dpf were used as initial conditions. Results were visualized using seaborn [74]. Error bars are the 95% confidence intervals of the measurement data and are calculated by seaborn. Relevant scripts, the PEtab problem and parameter estimation results are deposited on DaRUS (10.18419/darus-3838).

### Scanning electron microscopy

The fixed samples were critical point dried using a Leica CPD 300 critical point dryer.

Subsequently they were mounted on specimen mounts (diameter 25.4 mm, Plano, G399F) using double side sticking conductive pads (diameter 25 mm, Plano, G3348) and sputtered with Platinum using a Polaron Sputter Coater SC7640. For imaging a Zeiss Supra 40VP Field Emission SEM was used.

## Supplementary information

Supplemental Material

## Data Availability

For mathematical modelling, relevant scripts, the PEtab problem and parameter estimation results are deposited on DaRUS (10.18419/darus-3838).
