# Supplementary material for: A sphingolipid rheostat controls apoptosis versus apical cell extrusion as alternative tumour-suppressive mechanisms
Source: Cell Death Dis. 2024 Oct 14;15(10):746. doi: 10.1038/s41419-024-07134-2 (PMC11471799; doi:10.1038/s41419-024-07134-2)
Supplement: Supplementary file 1 — Supplemental Material [file 41419_2024_7134_MOESM1_ESM.docx]

**Supplementary Data**

The following file contains supplementary information for the paper “A sphingolipid rheostat controls apoptosis versus apical cell extrusion as alternative tumour-suppressive mechanisms”.

The file is composed of:

Supplementary Figures and related Figure legends (5 Figures)

Supplementary Table (1 Table)

Supplementary Notes (2 Notes)

**
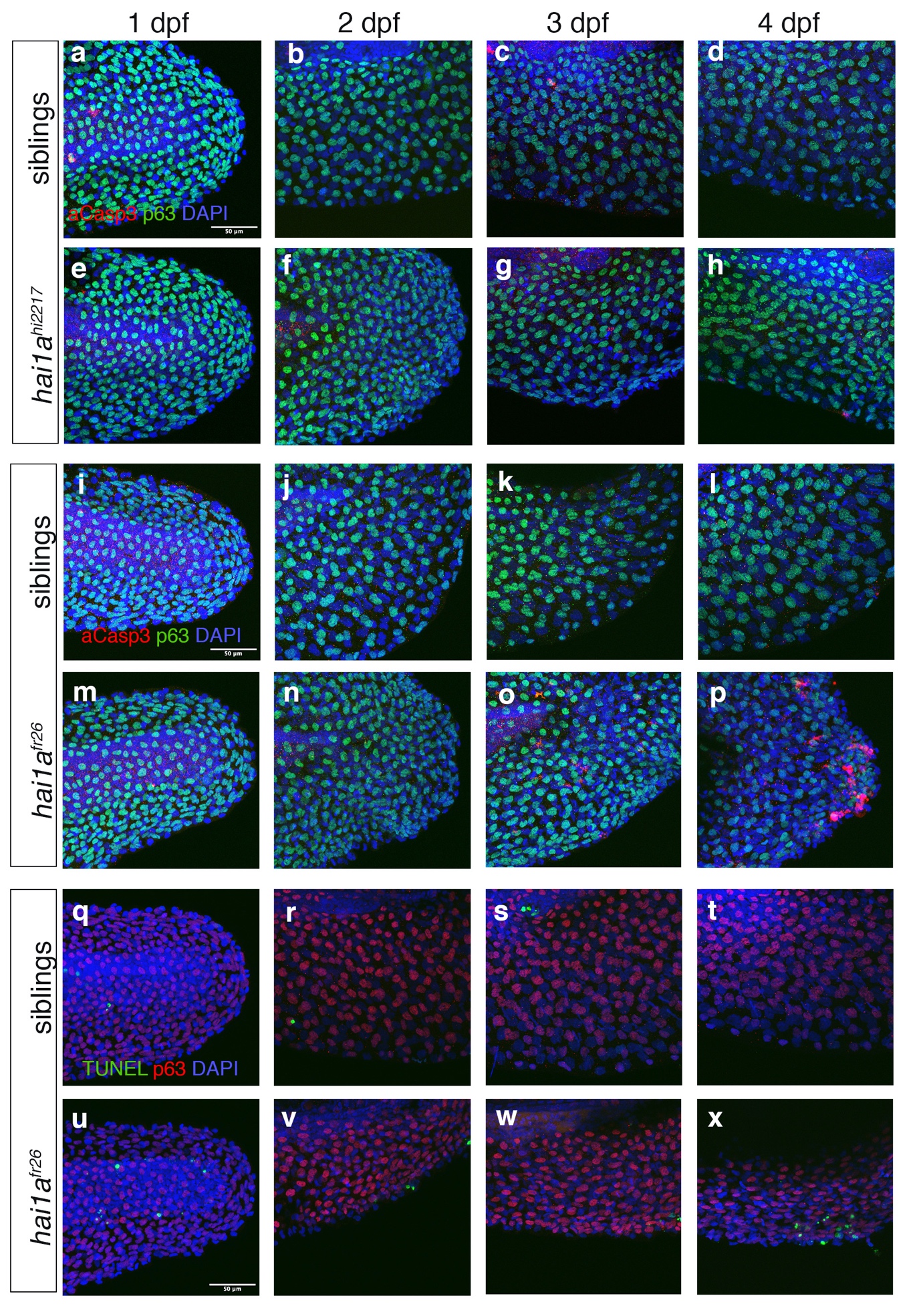
**

**Supplementary Figure S1 Time course of cell death in *hai1a^hi2217^* and *hai1a^fr26^* mutants**

**(a-h)** aCasp3-positive cells in the caudal fin fold of 1-4 dpf sibling control (a-d) or *hai1a^hi2217^* (e-h) mutants.

**(i-p)** aCasp3-positive cells in the caudal fin fold of 1-4 dpf sibling control (i-l) or *hai1a^fr26^* (m-p) mutants.

**(q-x)** TUNEL-positive cells in the caudal fin fold of 1-4 dpf sibling control (q-t) or *hai1a^fr26^* (u-x) mutants.

In (a-p) apoptotic cells are labeled with aCasp3 (red), basal keratinocytes with p63 (green), and nuclei using DAPI (blue). In (q-x) apoptotic cells are labeled with TUNEL (green), basal keratinocytes with p63 (red), and nuclei using DAPI (blue). Scale bars = 50 µm.

**
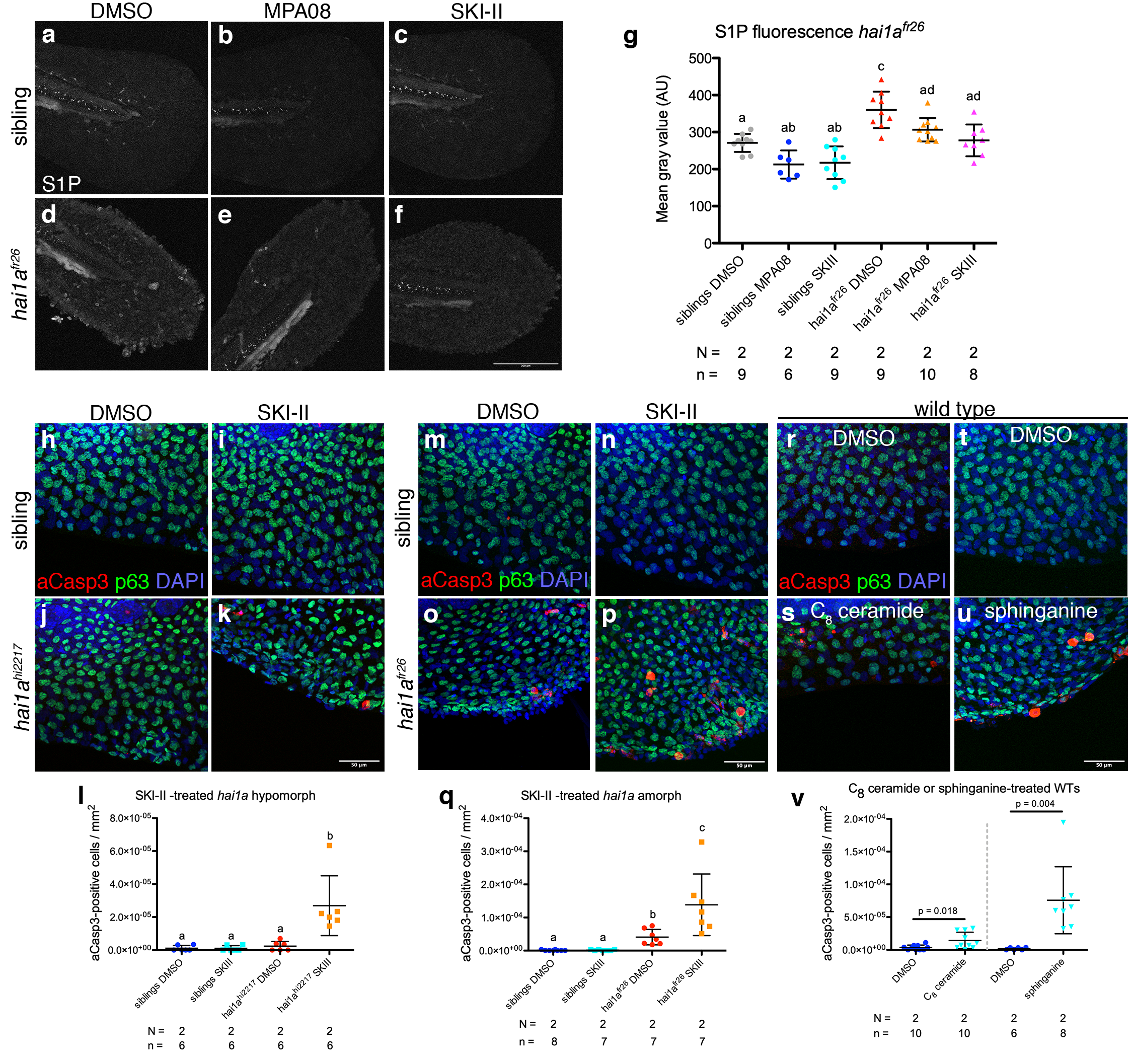
**

**Supplementary Figure S3 Sphingolipids modulate cell death in *hai1a* mutants**

**(a-f)** Anti-S1P whole mount immunostaining showing the caudal fin fold of 4 dpf *hai1a^fr26^* mutants, without (a,d) and with sphingosine kinase inhibition with either MPA08 (b,e) or SKI-II (c,f). Scale bar = 200 μm.

**(g)** Quantification of S1P fluorescence in the caudal fins of embryos, normalised to fin area.

**(h-k)** Apoptotic cells in the caudal fin fold of 4 dpf *hai1a^hi2217^* hypomorphic mutant embryos upon sphingosine kinase inhibition with SKI-II.

**(l)** Quantification of numbers of aCasp3-positive cells in the tail fins of embryos, normalised to fin area.

**(m-p)** Apoptotic cells in the caudal fin fold of 4 dpf *hai1a^fr26^* amorphic mutant embryos upon sphingosine kinase inhibition with SKI-II.

**(q)** Quantification of numbers of aCasp3-positive cells in the tail fins of embryos, normalised to fin area.

**(r-u)** Apoptotic cells in the caudal fin fold of 4 dpf wild-type embryos upon C_8_ ceramide (r-s) or sphinganine (t-u) treatment. C_8_ ceramide was complexed with fatty acid-free bovine serum albumin before treatment.

**(v)** Quantification of numbers of aCasp3-positive cells in the tail fins of embryos, normalised to fin area.

In (h-k, m-p, r-u) apoptotic cells are labeled with aCasp3 (red), basal keratinocytes with p63 (green), and nuclei using DAPI (blue). Scale bars = 50 µm. For (g, l, q) means were compared using one-way ANOVA with post-hoc Tukey’s multiple comparison test. For (v), means within each treatment group were compared using an unpaired two-tailed Student’s t-test. N = number of biological replicates, n = number of fish per condition.


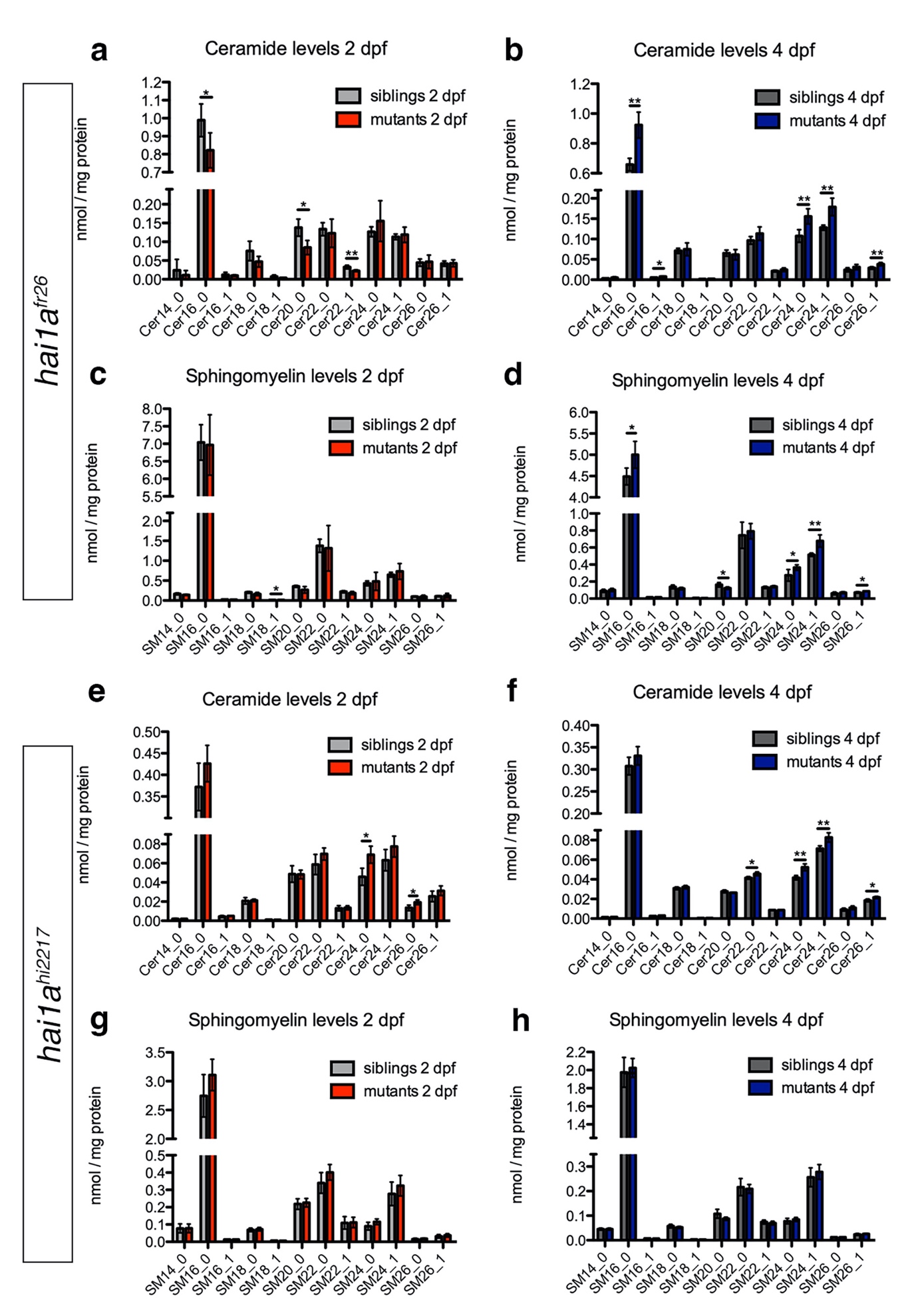


**Supplementary Figure S4 Lipidomics results overview**

Overview of lipidomics results for all ceramide and sphingomyelin species at 2 dpf (red bars) and 4 dpf (blue bars) in amorphic allele *hai1a^fr26^* **(a-d)** or hypomorphic allele *hai1a^hi2217^* **(e-h)**, compared to their respective sibling controls. Acyl chain lengths of the lipid species are indicated on the x axis below the graph. Bars represent the mean of N = 4 biological replicates per condition, with n = 300 fish per replicate; error bars represent standard deviation. Means of siblings and mutants within each time point were compared using an unpaired two-tailed Student’s t-test.

**
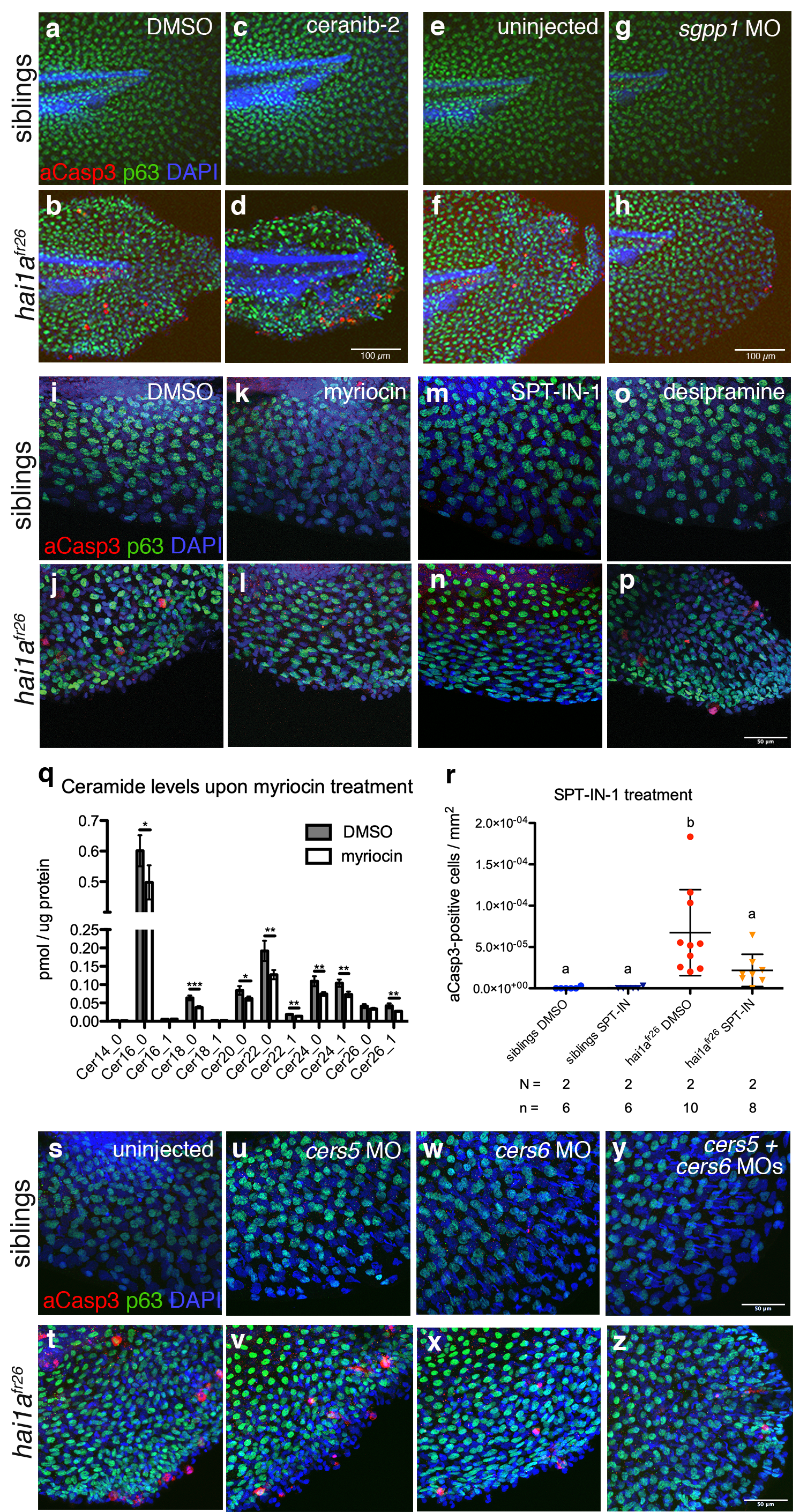
**

**Supplementary Figure S5 Alteration of epidermal cell death rates in *hai1a^fr26^* mutants upon manipulation of the rheostat**

Representative images of apoptotic cells in the caudal fin fold of 4 dpf sibling control and *hai1a^fr26^* mutant embryos upon:

**(a-d)** inhibition of ceramidases by ceranib-2 treatment, **(e-h)** s*gpp1* knockdown by MO injection,

**(i-p)** inhibition of *de novo* ceramide synthesis by myriocin or SPT-IN-1 treatment from 3-4 dpf or of sphingomyelinases by desipramine treatment.

**(q)** Lipidomics results for all ceramide species at 3 dpf in WT fish treated with DMSO (grey bars) or serine palmitoyltransferase inhibitor myriocin (white bars). Acyl chain lengths of the lipid species are indicated on the x axis below the graph. Bars represent the mean of N = 4 biological replicates per condition, with n = 300 fish per replicate; error bars represent standard deviation. Means of control and inhibitor-treated embryos within each ceramide species were compared using an unpaired two-tailed Student’s t-test.

**(r)** Quantification of numbers of aCasp3-positive cells in the tail fins of embryos treated with serine palmitoyltransferase inhibitor SPT-IN-1, normalised to fin area. Means were compared using one-way ANOVA with post-hoc Tukey’s multiple comparison test.

**(s-z)** Apoptotic cells in the caudal fin fold of 4 dpf sibling control and *hai1a^fr26^* mutant embryos upon inhibition of C_16_ ceramide production by *cers5* and/or *cers6* MO injection.

Apoptotic cells are labeled with aCasp3 (red), basal keratinocytes with p63 (green), and nuclei using DAPI (blue). Scale bar in a-h = 100 μm and in i-p and s-z = 50 μm. For quantification of aCasp3 cells aside from SPT-IN-1 treatment, see Fig. 5.


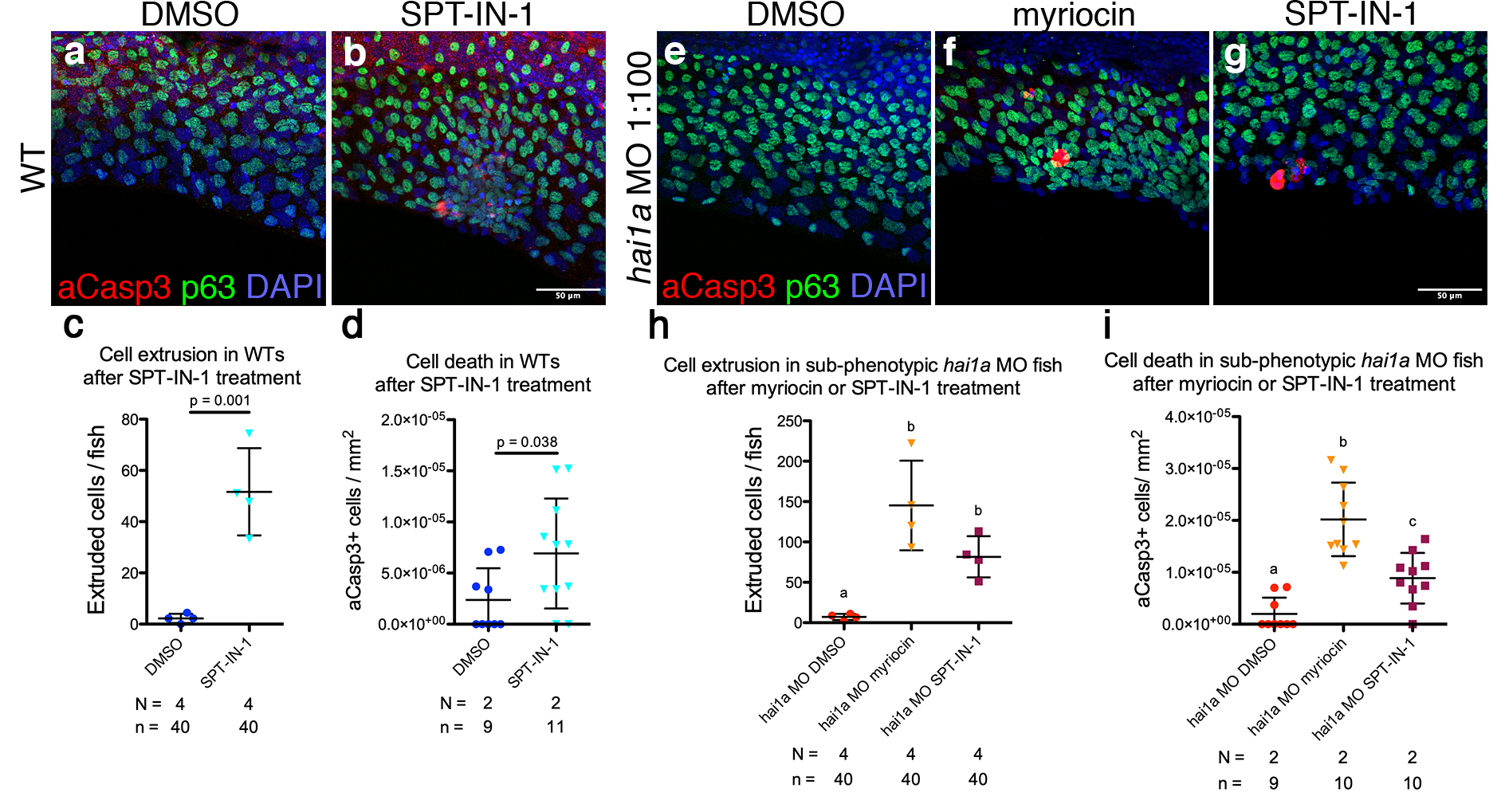


**Supplementary Figure S6 Apical cell extrusion and apoptosis upon early transient serine palmitoyltransferase inhibition**

**(a-b)** Apoptotic cells in the caudal fin fold of 4 dpf WT embryos upon serine palmitoyltransferase inhibition with SPT-IN-1 at 2 dpf, followed by inhibitor washout.

**(c)** Quantification of the numbers of extruded cells collected per WT fish at 4 dpf upon inhibition of *de novo* sphingolipid synthesis by SPT-IN-1 treatment at 2 dpf.

**(d)** Quantification of numbers of aCasp3-positive cells in the tail fins of embryos, normalised to fin area.

**(e-g)** Apoptotic cells in the caudal fin fold of 4 dpf embryos injected at the 1-cell stage with sub-phenotypic doses of *hai1a* morpholino, upon serine palmitoyltransferase inhibition with myriocin or SPT-IN-1 at 2 dpf, followed by inhibitor washout.

**(h)** Quantification of the numbers of extruded cells collected per *hai1a* morphant fish at 4 dpf upon inhibition of *de novo* sphingolipid synthesis by myriocin or SPT-IN-1 treatment at 2 dpf.

**(i)** Quantification of numbers of aCasp3-positive cells in the tail fins of embryos, normalised to fin area.

Apoptotic cells are labeled with aCasp3 (red), basal keratinocytes with p63 (green), and nuclei using DAPI (blue). Scale bars = 50 μm. Means in (c-d) were compared using an unpaired, two-tailed Student’s t-test, and in (h-i) by one-way ANOVA with post-hoc Tukey’s multiple comparison test.

**Supplementary Table 1:**

MRM transitions and compound-specific parameters for the MS detection of sphingoid bases

| **Q1 Mass (Da)** | **Q3 Mass (Da)** | **ID** | **DP (volts)** | **EP (volts)** | **CE (volts)** | **CXP (volts)** | **Quantifier** |
| --- | --- | --- | --- | --- | --- | --- | --- |
| 286.4 | 238.4 | sphingosine (d17:1) | 56 | 10 | 25 | 14 |  |
| 286.4 | 250.4 | sphingosine (d17:1) | 56 | 10 | 20 | 14 |  |
| 286.4 | 268.4 | sphingosine (d17:1) | 56 | 10 | 17 | 14 | X |
| 288.4 | 60.0 | sphinganine (d17:0) | 126 | 10 | 43 | 8 |  |
| 288.4 | 240.4 | sphinganine (d17:0) | 126 | 10 | 29 | 8 |  |
| 288.4 | 270.4 | sphinganine (d17:0) | 126 | 10 | 19 | 8 | X |
| 300.7 | 252.4 | sphingosine (d18:1) | 96 | 10 | 23 | 8 |  |
| 300.7 | 264.4 | sphingosine (d18:1) | 96 | 10 | 25 | 8 |  |
| 300.7 | 282.4 | sphingosine (d18:1) | 96 | 10 | 21 | 8 | X |
| 302.7 | 60.0 | sphinganine (d18:0) | 126 | 10 | 43 | 8 |  |
| 302.7 | 254.4 | sphinganine (d18:0) | 126 | 10 | 29 | 8 |  |
| 302.7 | 284.4 | sphinganine (d18:0) | 126 | 10 | 19 | 8 | X |

**Supplementary Note 1:** Metabolic Control Analysis (MCA)

We have generated a simplified model of the sphingolipid rheostat.

**
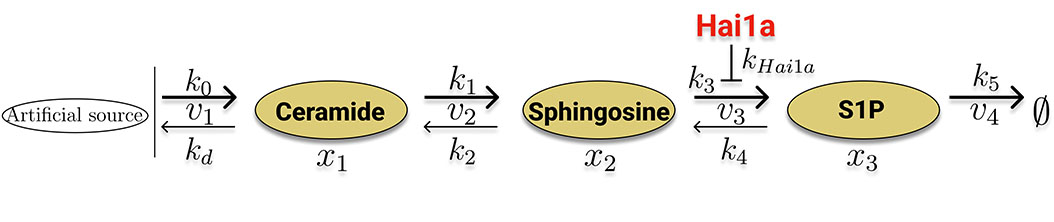
**

**Scheme 1** of simplified model of sphingolipid rheostat (also shown as Figure 4h of the main manuscript)

Model variables are concentrations of ceramide ($x_{1}$), sphingosine ($x_{2}$) and S1P ($x_{3}$), which are connected via four reactions $v_{1}$, …, $v_{4}$. The first three are reversible, $v_{4}$ is irreversible. Ceramide synthesis pathways are described as a single effective constant influx in the model. The dynamic of this system is described by a differential equation system and reads

| $\dot{x}= Sv\left( x \right)=\left( \begin{matrix} 1 & -1 & 0 & 0 \\ 0 & 1 & -1 & 0 \\ 0 & 0 & 1 & -1 \end{matrix} \right)\left( \begin{matrix} v_{1}\left( x \right) \\ v_{2}\left( x \right) \\ v_{3}\left( x \right) \\ v_{4}\left( x \right) \end{matrix} \right),$ | (1) |
| --- | --- |

with stochiometric matrix $S\in\mathbb{R}^{3\times4}$ and flux vector $v\left( x \right)\in\mathbb{R}^{4}$. The system does not obey any conservation relations and eventually reaches a dynamic steady state with a steady state net flux $J=\alpha\left( 1,1,1,1 \right)^{t}$to the right. The steady state concentrations can be obtained by solving $Sv\left( \overline{x} \right)=0$ for $\overline{x}$. Calculation of $\overline{x}$ requires defining reaction rate equations, which can be done by using mass action kinetics,

| $v_{1}\left( x \right)=k_{0}-k_{d}x_{1}$  $v_{2}\left( x \right)=k_{1}x_{1}-k_{2}x_{2}$  $v_{3}\left( x \right)=k_{3}x_{2}-k_{4}x_{3}$  $v_{4}\left( x \right)=k_{5}x_{3}.$ | (2) |
| --- | --- |

The steady-state concentrations of the three species $\overline{x}_{i}$ depend thereby on the values of the unknown rate constants $k_{i}$, which can be summarized in a parameter vector $\theta$,

$$\theta=\left( k_{0}, k_{d}, k_{1}, k_{2}, k_{3}, k_{4}, k_{5} \right)\in\mathbb{R}_{>0}^{7}.$$

Solving for $\overline{x}$ is possible here and leads to

$$\overline{x}_{1}\left( \theta\right)=\frac{k_{0}\left( k_{2}\left( k_{4}+k_{5} \right)+k_{3}k_{5} \right)}{k_{d}k_{2}(k_{4}+k_{5})+k_{d}k_{3}k_{5} + k_{1}k_{3}k_{5}}$$

$$\overline{x}_{2}\left( \theta\right)=\frac{k_{0}k_{1}\left( k_{4}+k_{5} \right)}{k_{d}k_{2}(k_{4}+k_{5})+k_{d}k_{3}k_{5} + k_{1}k_{3}k_{5}}$$

$$\overline{x}_{3}\left( \theta\right)=\frac{k_{0}k_{1}k_{3}}{k_{d}k_{2}(k_{4}+k_{5})+k_{d}k_{3}k_{5} + k_{1}k_{3}k_{5}}.$$

Metabolic Control Analysis (MCA) ^36,37^ was applied to investigate if the observed fold changes in lipid concentrations are in accordance with the sphingolipid rheostat model. MCA quantifies (local) sensitivities of steady-state properties of metabolic reaction networks to parameter changes. Since this theory linearizes the system about its steady state, quantitative statements about the coefficients can only be made if the parameter changes are sufficiently small. However, we anticipate that qualitative statements about signs of coefficients also persist for larger perturbations and are independent of the exact choice of reaction rate laws, allowing a qualitative investigation of the long-term sphingolipid rheostat behaviour via MCA.

For the MCA, we consider the sphingolipid rheostat in its dynamic steady state for each condition. Since the embryos are growing over the investigated period, such a steady state in lipid concentrations is achieved if the number of lipids increases accordingly. The concentration control coefficients $C_{v_{k}}^{\overline{x}_{i}}$

$$C_{v_{k}}^{\overline{x}_{i}}=\frac{v_{k}}{\overline{x}_{i}}\frac{\partial\overline{x}_{i}}{\partial v_{k}}$$

indicate changes in the steady-state concentrations $\overline{x}_{i}$ upon changes in the rate $v_{k}$ of reaction $k$. The sign of the concentration control coefficient determines whether a change in the reaction flux increases or decreases the steady-state concentration. Therefore, a positive control coefficient $C_{v_{k}}^{\overline{x}_{i}}$ corresponds to a fold-change greater than one for metabolite species $i$ when the flux $v_{k}$ is increased.

For larger networks with $r$ reactions and $n$metabolic species, concentration control coefficients are collected in a concentration control coefficient matrix $C^{C}$ of dimension $n\times r$,

$$C^{C}=-\left( dg\overline{x} \right)^{-1}\left( \left( S\left. \frac{\partial v\left( x \right)}{\partial x} \right|_{x=\overline{x}} \right)^{-1}S \right)\left( dgJ \right),$$

with diagonal matrices $dgJ$ and $dg\overline{x}$ of dimensions $r\times r$ and $n\times n$ that contain the steady state fluxes $J_{j}$ and steady-state concentrations $\overline{x}_{i}$ on the diagonals.

The *hai1a^fr26^* mutation increases the conversion from sphingosine to S1P by uncontrolled Matriptase activity, which is reflected in our model by an increase in the rate constant $k_{3}$ in the mutant compared to the sibling controls. The concentration control coefficients $C_{v_{3}}^{\overline{x}_{1}}, C_{v_{3}}^{\overline{x}_{2}}, C_{v_{3}}^{\overline{x}_{3}}$ describe the relative changes in the steady-state concentrations of the three lipids ceramide, sphingosine, and S1P upon a change in reaction 3 (5^th^ column in the sign matrix $\sigma(C^{C});$see below). Calculation of the concentration control coefficient matrix for a specific parameter choice is demonstrated in Materials and Methods.

Importantly, the sign structure of the control coefficient matrix $C^{C}$is independent of the values of the rate constants $\theta$, as can be seen by calculating the derivatives of $\overline{x}$ with respect to $\theta$. In particular, the sign of $C_{v_{3}}^{\overline{x}_{1}}$ is given by the sign of the derivative of $\overline{x}_{1}$ with respect to rate constant $k_{3}$,

$$\frac{d\overline{x}_{1}\left( \theta\right)}{dk_{3}}=\frac{-k_{0}k_{2}\left( k_{4}+k_{5} \right)k_{1}k_{5}}{\left( k_{d}k_{2}\left( k_{4}+k_{5} \right)+k_{d}k_{3}k_{5} + k_{1}k_{3}k_{5} \right)^{2}}<0.$$

Our result can be generalized to changes of all fluxes and metabolites in the rheostat, as indicated by the sign structure $\sigma(C^{C})$of the control coefficient matrix:


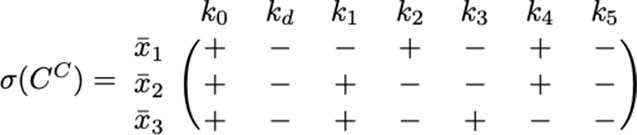


As $C_{v_{3}}^{\overline{x}_{1}}$ is negative, the corresponding fold-change of ceramide must be lower than 1, implying that the observed increase in ceramide in the mutant at 4 dpf cannot be explained with the current model structure. In summary, our MCA approach shows that the ceramide over-production observed in the *hai1a^fr26^* mutants at 4 dpf is not consistent with the reaction network structure of the rheostat as illustrated in Scheme 1.

**Supplementary Note 2:** Ordinary Differential Equation (ODE) model

Compared to the analysis described in Supplementary Note 1, we first extended the rheostat model (Equation 1) to sphinganine to include all available experimental lipid data. The revised model includes the additional flux of sphinganine synthesis $v_{0}$ and is described by

| $\dot{x}= Sv(x) =\left( \begin{matrix} \boldsymbol{1} & \boldsymbol{-1} & \boldsymbol{0} & \boldsymbol{0} & \boldsymbol{0} \\ \boldsymbol{0} & 1 & -1 & 0 & 0 \\ \boldsymbol{0} & 0 & 1 & -1 & 0 \\ \boldsymbol{0} & 0 & 0 & 1 & -1 \end{matrix} \right)\left( \begin{matrix} \boldsymbol{v}_{\boldsymbol{0}}\boldsymbol{(x)} \\ v_{1}(x) \\ v_{2}(x) \\ v_{3}(x) \\ v_{4}(x) \end{matrix} \right)$ | (3) |
| --- | --- |

with stoichiometric matrix $S\in\mathbb{R}^{4\times5}$ and flux vector $v\left( x \right)\in\mathbb{R}^{5}$. The additional state variable and the revised flux equation are indicated in bold.

Second, we considered a negative feedback loop in which the concentration of ceramide controls its own synthesis. This model postulates that the synthesis of ceramide is promoted when a ceramide concentration below a certain threshold is detected at 2 dpf. This synthesis could lead to an excessive production at 4 dpf if its deactivation does not take place quickly enough.


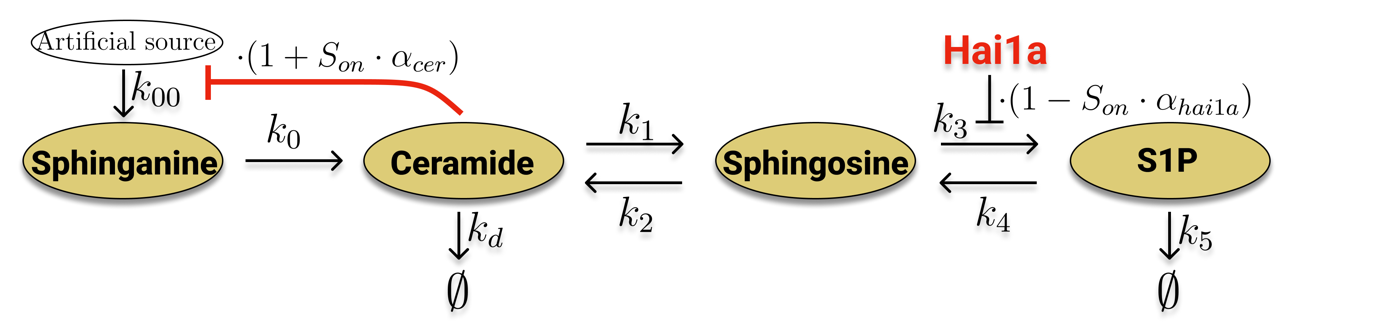


**Scheme 2** (also shown as Figure 4i of the main manuscript), incorporating experimental sphinganine data and considering de novo ceramide synthesis from sphinganine, as well as negative feedback from ceramide to *de novo* synthesis via sphinganine (k_00_)

To account for the different impacts of Hai1a and Matriptase on sphingosine kinase activity and the ceramide - S1P flux in *hai1a^fr26^* mutants versus wild types, a Boolean operator $S_{on}$ was used that was set active ${(S}_{on}=1)$ in the wild type and inactive ${(S}_{on}=0)$ in the mutant. The apparent S1P synthesis rate in the wild type was set at least 50% lower than in the mutant $k_{3, app}=k_{3}\cdot\left( 1-{S_{on}\cdot\alpha}_{hai1a} \right), 0.5\leq\alpha_{hai1a}<1$. In addition, for the proposed negative feedback, $S_{on}$ was again used to distinguish wild type and mutant, and the *de novo* lipid synthesis rate was modulated by ceramide levels so that the feedback is only active when ceramide is above a threshold: $k_{00,app}=k_{00}\cdot{(1+S_{on}\cdot\alpha}_{cer}), -1<\alpha_{cer}<0$.

The ODE-System of the computational model reads

| $\frac{d \mathrm{Sphinganine}}{dt}=k_{00}\cdot\left( 1+S_{on}\cdot\alpha_{cer} \right)-k_{0}\cdot\mathrm{Sphinganine}$  $\frac{d \mathrm{Ceramide}}{dt}=k_{0}\cdot\mathrm{Sphinganine}+k_{2}\cdot\mathrm{Sphingosine}-\left( k_{1}+kd \right)\cdot\mathrm{Ceramide}$  $\frac{d \mathrm{Sphingosine}}{dt}=k_{1}\cdot\mathrm{Ceramide}+k_{4}\cdot S1p-\left( k_{2}+k_{3}\left( 1-S_{on}\cdot\alpha_{hai1a} \right) \right)\cdot\mathrm{Sphingosine}$  $\frac{d S1P}{dt}=k_{3}\left( 1-S_{on}\cdot\alpha_{hai1a} \right)\cdot\mathrm{Sphingosine}-\left( k_{4}+k_{5} \right)\cdot S1P$ | (4) |
| --- | --- |

We used maximum likelihood estimation to fit the parameters of the revised model to experimental data (details of the optimization are given in Materials and Methods). Measurements at 2 dpf were used as initial conditions. At 4 dpf, the model can fit all lipid measurements and simulation results for sphinganine, sphingosine and ceramide lie perfectly within the 95% confidence intervals of the experimental data. Overall, the fits show that the negative feedback can explain the over-production of ceramide at 4 dpf in the mutant. In short, the revised model with the negative feedback sensing mechanism can capture the ceramide over-production in the *hai1a^fr26^* mutant at 4 dpf well.

To show that a computational model without negative feedback is not able to explain the experimental data, we tried to fit the model to the data without including negative feedback. To do this, the model was recalibrated using $\alpha_{cer}=0$ (i.e. no negative feedback was present). Respective model fits are qualitatively different from experimental observations. Here, the sphinganine, sphingosine and ceramide concentrations cannot all be higher in the mutant compared to the wild type. Parameters are estimated such that sphinganine concentrations in the mutant and in the wild type are equal at 4 dpf. Mimicking the observed ceramide overproduction at 4 dpf is not possible and the 95% confidence intervals of the experimental data cannot be met. The model prediction for ceramide in the wild type is also outside of the 95% confidence interval.
